# Supplementary material for: Different dose regimens of a SARS-CoV-2 recombinant spike protein vaccine (NVX-CoV2373) in younger and older adults: A phase 2 randomized placebo-controlled trial
Source: PLoS Med. 2021 Oct 1;18(10):e1003769. doi: 10.1371/journal.pmed.1003769 (PMC8486115; doi:10.1371/journal.pmed.1003769)
Supplement: S1 Text — Table A. Toxicity grading scales for solicited local and systemic adverse events—modified from FDA toxicity grading scale for clinical abnormalities. Table B. Adverse events of special interest relevant to COVID-19. Table C. Potential immune-mediated medical conditions. Fig A. Vaccine regimens and key trial assessments. Table D. Overall summary of solicited and unsolicited adverse events following vaccination of SARS-CoV-2 rS with Matrix-M1 adjuvant in adult participants 18 to 84 years of age (Safety Analysis Set). Table E. Percentage of all participants (18 to 84 years) experiencing solicited local and systemic adverse events by symptom, vaccination dose, vaccine group, and maximum toxicity grade (Safety Analysis Set). Table F. Percentage of younger adults (18 to 59 years) experiencing solicited local and systemic adverse events by symptom, vaccination dose, vaccine group, and maximum toxicity grade (Safety Analysis Set). Table G. Percentage of older adults (60 to 84 years) experiencing solicited local and systemic adverse events by symptom, vaccination dose, vaccine group, and maximum toxicity grade (Safety Analysis Set). Table H. Unsolicited adverse events by system organ class and preferred term reported in 1% or more participants in any group through 35 days after first vaccination (Safety Analysis Set). (DOCX) [file pmed.1003769.s002.docx]

**Supplementary Appendix**

This supplementary appendix has been provided by the authors to give readers additional information about their work.

S1 Text

Supplementary Appendix to Manuscript Entitled

**Different dose regimens of a SARS-CoV-2 recombinant spike protein vaccine (NVX-CoV2373) in younger and older adults: A phase 2 randomized placebo-controlled trial**

**Table of Contents**

2019nCoV-101 Study Group Members ………………………………………………………………..3

[Supplemental Methods 4](#_Toc72782448)

[Vaccination Pause Rules 4](#_Toc72782449)

[Immunologic Assay Method Details 5](#_Toc72782450)

[Table A. Toxicity Grading Scales for Solicited Local and Systemic Adverse Events – Modified From FDA Toxicity Grading Scale for Clinical Abnormalities 7](#_Toc72782451)

[Table B. Adverse Events of Special Interest Relevant to COVID-19 8](#_Toc72782452)

[Table C. Potential Immune-Mediated Medical Conditions. 9](#_Toc72782453)

Fig A. Vaccine Regimens and Key Trial Assessments …....………………………………….. 10

[Table D. Overall Summary of Solicited and Unsolicited Adverse Events Following Vaccination of SARS-CoV-2 rS With Matrix-M1 Adjuvant in Adult Participants 18 to 84 Years of Age (Safety Analysis Set) 11](#_Toc72782455)

[Table E. Percentage of All Participants (18 to 84 years) Experiencing Solicited Local and Systemic Adverse Events by Symptom, Vaccination Dose, Vaccine Group, and Maximum Toxicity Grade (Safety Analysis Set) 12](#_Toc72782456)

[Table F. Percentage of Younger Adults (18 to 59 Years) Experiencing Solicited Local and Systemic Adverse Events by Symptom, Vaccination Dose, Vaccine Group, and Maximum Toxicity Grade (Safety Analysis Set) 18](#_Toc72782457)

[Table G. Percentage of Older Adults (60 to 84 Years) Experiencing Solicited Local and Systemic Adverse Events by Symptom, Vaccination Dose, Vaccine Group, and Maximum Toxicity Grade (Safety Analysis Set) 24](#_Toc72782458)

[Table H. Unsolicited Adverse Events by System Organ Class and Preferred Term Reported in 1% or More Participants in Any Group Through 35 Days After First Vaccination (Safety Analysis Set) 2](#_Toc72782459)9

**2019nCoV-101 Study Group Members**

**(listed in PubMed, and ordered alphabetically by institutional affiliation)**

The following study group members were all closely involved with the design, implementation, and oversight of the phase 2 part of the NVX-CoV2373-2019nCoV-101 clinical trial.

Advanced Clinical Research, Meridian, Idaho, United States: Mark Turner

Alliance for Multispecialty Research, Wichita and Newton, Kansas, United States: Terry Klein, Richard Glover II

Australian Clinical Research Network, Maroubra, NSW, Australia: Mark Arya

Barwon Health, Geelong, Melbourne, Victoria, Australia: Eugene Athan

Central Kentucky Research Associates, Lexington, Kentucky, United States: Mark Adams

Meridian Clinical Research, Rockville, Maryland, and Savannah, Georgia, United States: Ira Berger, Paul Bradley

Novavax, Inc., Gaithersburg, Maryland, United States: Neil Formica, Raburn Mallory, Gary Albert, Michelle Robinson, Joyce S. Plested, Iksung Cho, Andreana Robertson, Filip Dubovsky, Gregory M. Glenn

Nucleus Network Pty Ltd, Melbourne, Victoria, and Herston, Queensland, Australia: Jason Lickliter, Paul Griffin

Paratus Clinical Research – Canberra, ACT, and Central Coast and Western Sydney, NSW, Australia: Amber Leah, Josha Kim, Fiona Napier-Flood

Rapid Medical Research, Cleveland, Ohio, United States: Mary Beth Manning

Scientia Clinical Research Limited, Randwick, NSW, Australia: Charlotte Lemech

Synexus Clinical Research US, Cincinnati, Ohio, United States: Paul Nugent

University of the Sunshine Coast, Sippy Downs and Morayfield, Queensland, Australia: Susan Thackwray, Scott Kitchener

# Supplemental Methods:

## Vaccination Pause Rules

Adverse events meeting any one of the following criteria will result in an immediate enrollment or further dosing hold for Part 2 of the study, pending further review by the Safety Monitoring Committee (SMC) at the direction of the SMC Chair:

- Any toxicity grade 3 (severe) solicited single adverse event term across all study vaccine groups (blinded) occurring in ≥5% of participants for either study age group (after a minimum of 100 participants are enrolled in either age group) following vaccination (first, second vaccinations and booster dose to be assessed separately).
- Any grade 3 (severe) unsolicited single adverse event preferred term for which the investigator assesses as related which occurs in ≥7 participants overall (both age groups) within 35 days following first-dose vaccination.

In addition, any serious adverse event assessed as related to vaccine (by study investigator and/or the sponsor) will be reported by the sponsor to the SMC Chair as soon as possible, and within 24 hours of the sponsor’s awareness of the event. Based on this initial report of the event to the SMC Chair, the Chair may advise the sponsor to immediately pause enrollment and further dosing in either some or all participants in the study and to convene an ad hoc meeting, or make alternative recommendations. The SMC Charter defines processes for how this review will occur and how the Chair’s recommendations will be documented.

The sponsor, along with medical monitor, may request an SMC review for any safety concerns that may arise in the trial and not associated with any specific pause rule.

## Immunologic Assay Method Details

SARS-CoV-2 Spike Protein Serum IgG ELISA. rSARS-CoV-2 vaccine protein was immobilized onto the surface of the 96-well microtiter plate wells (100 µL per well) by direct adsorption for 15 to 96 hours at 2°C to 8°C at a concentration of 1 µg/mL in PBS. Plates were washed 3 times with 300 µL/well PBST, blocked with 300 µL blocking buffer for 1 to 1.5 hours at 24°C ± 2°C. Diluted reference standard (2-fold dilution series of 12 dilutions starting 1:1000) and human serum samples (3-fold dilution series of 12 dilutions) in assay buffer (1% milk in PBS) starting at 1:100 dilution are then added in duplicate (100 µL per well) to the rSARS-CoV-2 vaccine protein-coated wells and any specific antibodies are allowed to complex with the coated antigen for 2 hours at 24°C ± 2°C. Plates are washed 2 times with 300 µL/well PBST. Antibodies bound to the rSARS-CoV-2 vaccine protein are then detected using a horseradish peroxidase (HRP) conjugate goat anti-human IgG antibody diluted 1:2000 (Southern Biotech cat no. 2040-05), incubated for 1 hour ± 10 minutes at 24°C ± 2°C, washed 3 times with 300 µL/well PBST and a colorimetric signal generated by addition of 100 µL per well 3, 3′,5,5′-tetramethylbenzidine (TMB) chromogenic substrate for 10 minutes ± 2 minutes at 24°C ± 2°C. After incubation was complete, the TMB reaction was stopped with 100 µL per well of TMB stop solution. The absorbance was measured at 450 nm using a molecular device 96-well plate reader. When binding reagents (coated antigen and secondary antibody) are in excess, the optical density (OD) of the chromogenic substrate at endpoint is proportional to the quantity of anti-rSARS-CoV-2 IgG present in the serum sample. The total anti-SARS-CoV-2 S protein IgG antibody level in a serum sample was quantitated in ELISA unit, EU/mL by comparison to a reference standard curve. The results were analyzed by SoftMax Pro software using 4-PL curve fit. Assay included control plates comprising positive controls and negative controls.

SARS-CoV-2 Microneutralization Assay. The viral working stock was SARS-CoV-2 hCoV-19/Australia/VIC01/2020 (GenBank MT007544.1) and was a gift from Melbourne’s Peter Doherty Institute for Infection and Immunity (Melbourne, Australia). Sequence analysis of the SARS-CoV-2 hCoV-19/Australia/VIC01/2020 spike protein shows high sequence homology with the prototype strain with only one nucleotide difference in the spike protein (S247R) compared to the prototype strain. All serum samples were heat inactivated at 56°C for 30 minutes to remove complement and allowed to equilibrate to room temperature prior to processing for neutralization titer. Sera were diluted by addition of 28 µL to 252 µL of assay media in a 96-well dilution plate and an 11-point 2-fold serial dilution was prepared. Sera were then mixed with an equal volume (140 µL) of SARS-CoV-2 (4000 TCID_50_ units/mL) and incubated for 1 hour at 37^o^C, 5% CO_2_. The MN assay media (AM) comprised Dulbecco Essential Medium without L-glutamine (DMEM; Thermo Fisher Scientific, cat. no. 10313-021) supplemented with 2% FBS (Bovagen SFBS), 1% Glutamax (Thermo Fisher Scientific, cat. no. 35050-061), and 1% Pen/Strep (Thermo Fisher Scientific, cat. no. 15140-122).

Following this incubation, 100 µL of the virus/serum mixtures (100 TCID_50_ units/well) were added in duplicate to Vero E6 cells, pre-seeded 24 hours prior in 96-well plates in 100 µL of assay media at 1.5 × 104 cells/well plates, and were incubated for 3 days at 37^o^C, 5% CO_2_.

The residual non-neutralized virus is detected via cytopathic effect (CPE) by microscopic scoring. Two replicate wells per dilution are scored as either positive (SARS-CoV-2 cytopathology is present) or negative (healthy Vero E6 monolayer). The neutralization titer is expressed as the reciprocal of the highest dilution at which 50% of the replicate wells are protected from infection (MN_50_).

The final serum dilution range of the assay was MN_50_ titer range of 20‒20,480. If at least 50% protection was not observed for an individual serum at any dilution, the MN_50_ titer was recorded as <20. Not less than 4 virus control only wells, and mock infected wells (no virus) were run per plate.

### Table A. Toxicity Grading Scales for Solicited Local and Systemic Adverse Events – Modified From FDA Toxicity Grading Scale for Clinical Abnormalities.

| Local Reaction to Injectable Product | Mild (Grade 1) | Moderate  (Grade 2) | Severe (Grade 3) | Potentially Life Threatening  (Grade 4) |
| --- | --- | --- | --- | --- |
| Pain | Does not interfere with activity | Repeated use of non-prescription pain reliever >24 hours or interferes with activity | Significant; any use of prescription pain reliever or prevents daily activity | Requires ER visit or hospitalization |
| Tenderness | Mild discomfort to touch | Discomfort with movement | Significant discomfort at rest | Requires ER visit or hospitalization |
| Erythema/redness* | 2.5 − 5 cm | 5.1 − 10 cm | >10 cm | Necrosis or exfoliative dermatitis† |
| Induration/swelling* | 2.5 − 5 cm | 5.1 − 10 cm | >10 cm | Necrosis† |
| **Systemic (General)** | | | | |
|  | **Mild (Grade 1)** | **Moderate (Grade 2)** | **Severe (Grade 3)** | **Potentially Life Threatening (Grade 4)** |
| Fever‡ (°C)  (°F) | 38.0 − 38.4 100.4 − 101.1 | 38.5 − 38.9 101.2 − 102.0 | 39.0 − 40 102.1 − 104 | >40 >104 |
| Nausea/vomiting | Does not interfere with activity or 1 − 2 episodes/24 hours | Some interference with activity or >2 episodes/24 hours | Prevents daily activity, or requires IV hydration outside of hospital | Requires ER visit or hospitalization |
| Headache | Does not interfere with activity | Repeated use of non-prescription pain reliever >24 hours or interferes with activity | Significant; any use of prescription pain reliever or prevents daily activity | Requires ER visit or hospitalization |
| Fatigue/Malaise | Does not interfere with activity | Some interference with activity | Significant, prevents daily activity | Requires ER visit or hospitalization |
| Myalgia | Does not interfere with activity | Some interference with activity | Significant, prevents daily activity | Requires ER visit or hospitalization |
| Arthralgia | Does not interfere with activity | Some interference with activity | Significant, prevents daily activity | Requires ER visit or hospitalization |
| Abbreviations: ER = emergency room; IV = intravenous.  * The measurements should be recorded as a continuous variable.  † These events are not subject reported through the electronic diary and will be monitored through the adverse event pages of the study database.  ‡ Oral temperature if subject collected, sites may collect temperature using local clinic practices/devices. Toxicity grade will be derived. | | | | |

| Table B. Adverse Events of Special Interest Relevant to COVID-19.* | |
| --- | --- |
| **Body System** | **Diagnoses** |
| Immunologic | Enhanced disease following immunization, cytokine release syndrome related to COVID-19 disease,† multisystem inflammatory syndrome in children (MIS-C) |
| Respiratory | Acute respiratory distress syndrome (ARDS) |
| Cardiac | Acute cardiac injury including:   - Microangiopathy - Heart failure and cardiogenic shock - Stress cardiomyopathy - Coronary artery disease - Arrhythmia - Myocarditis, pericarditis |
| Hematologic | Coagulation disorder   - Deep vein thrombosis - Pulmonary embolus - Cerebrovascular stroke - Limb ischemia - Hemorrhagic disease - Thrombotic complications |
| Renal | Acute kidney injury |
| Gastrointestinal | Liver injury |
| Neurologic | Guillain Barré syndrome, anosmia, ageusia, meningoencephalitis |
| Dermatologic | Chilblain-like lesions, single organ cutaneous vasculitis, erythema multiforme |
| * COVID-19 disease manifestations associated with more severe presentation and decompensation with consideration of enhanced disease potential. The current listing is based on Safety Platform for Emergency Vaccines (SPEAC) D2.3 Priority List of Adverse Events of Special Interest: COVID-19 (SPEAC 2020).  † Cytokine release syndrome related to COVID-19 disease is a disorder characterized by nausea, headache, tachycardia, hypotension, rash, and/or shortness of breath. | |
|  | |

| Table C. Potential Immune-Mediated Medical Conditions. | |
| --- | --- |
| **Categories** | **Diagnoses (as MedDRA Preferred Terms)** |
| Neuroinflammatory Disorders: | Acute disseminated encephalomyelitis (including site specific variants: eg, non-infectious encephalitis, encephalomyelitis, myelitis, myeloradiculomyelitis), cranial nerve disorders including paralyses/paresis (eg, Bell’s palsy), generalized convulsion, Guillain-Barré syndrome (including Miller-Fisher syndrome and other variants), immune-mediated peripheral neuropathies and plexopathies (including chronic inflammatory demyelinating polyneuropathy, multifocal motor neuropathy, and polyneuropathies associated with monoclonal gammopathy), myasthenia gravis, multiple sclerosis, narcolepsy, optic neuritis, transverse myelitis, uveitis |
| Musculoskeletal and Connective Tissue Disorders: | Antisynthetase syndrome, dermatomyositis, juvenile chronic arthritis (including Still’s disease), mixed connective tissue disorder, polymyalgia rheumatic, polymyositis, psoriatic arthropathy, relapsing polychondritis, rheumatoid arthritis, scleroderma (including diffuse systemic form and CREST syndrome), spondyloarthritis (including ankylosing spondylitis, reactive arthritis [Reiter's syndrome] and undifferentiated spondyloarthritis), systemic lupus erythematosus, systemic sclerosis, Sjogren’s syndrome |
| Vasculitides: | Large vessel vasculitis (including giant cell arteritis such as Takayasu's arteritis and temporal arteritis), medium sized and/or small vessel vasculitis (including polyarteritis nodosa, Kawasaki's disease, microscopic polyangiitis, Wegener's granulomatosis, Churg–Strauss syndrome [allergic granulomatous angiitis], Buerger’s disease [thromboangiitis obliterans], necrotizing vasculitis, and anti-neutrophil cytoplasmic antibody [ANCA] positive vasculitis [type unspecified], Henoch-Schonlein purpura, Behcet's syndrome, leukocytoclastic vasculitis) |
| Gastrointestinal Disorders: | Crohn’s disease, celiac disease, ulcerative colitis, ulcerative proctitis |
| Hepatic Disorders: | Autoimmune hepatitis, autoimmune cholangitis, primary sclerosing cholangitis, primary biliary cirrhosis |
| Renal Disorders: | Autoimmune glomerulonephritis (including IgA nephropathy, glomerulonephritis rapidly progressive, membranous glomerulonephritis, membranoproliferative glomerulonephritis, and mesangioproliferative glomerulonephritis |
| Cardiac Disorders: | Autoimmune myocarditis/cardiomyopathy |
| Skin Disorders: | Alopecia areata, psoriasis, vitiligo, Raynaud’s phenomenon, erythema nodosum, autoimmune bullous skin diseases (including pemphigus, pemphigoid and dermatitis herpetiformis), cutaneous lupus erythematosus, morphoea, lichen planus, Stevens-Johnson syndrome, Sweet’s syndrome |
| Hematologic Disorders: | Autoimmune hemolytic anemia, autoimmune thrombocytopenia, antiphospholipid syndrome, thrombocytopenia |
| Metabolic Disorders: | Autoimmune thyroiditis, Grave’s or Basedow’s disease, Hashimoto thyroiditis,^*^ diabetes mellitus type 1, Addison’s disease |
| Other Disorders: | Goodpasture syndrome, idiopathic pulmonary fibrosis, pernicious anemia, sarcoidosis |
| Abbreviation: MEDRA = *Medical Dictionary for Regulatory Activities,* version 23.0.  * For Hashimoto’s thyroiditis: new onset only. | |

[FIG A APPEARS AFTER TABLE C]

[FIG A -- appears after Table C – FIG A is in Word, thus appears in S1_Text]

**Fig A. Vaccine Regimens and Key Trial Assessments.**

Shown are the planned randomization schema and associated vaccine regimens administered in the trial (Panel A in figure), along with timing of the key safety and immunogenicity assessments (Panel B in figure).

| **A: Vaccine Regimens** | | | | | | | | | | | |
| --- | --- | --- | --- | --- | --- | --- | --- | --- | --- | --- | --- |
| **Vaccine**  **Group** | **No. of Participants** | | **Day 0** | | **Day 21**  **–1 to +3 days** | |  | **Vaccine Group** | **Day 189**  **±15 days** | |  |
|  | Randomized | | rSARS-CoV-2 | Matrix-M1  adjuvant | rSARS-CoV-2 | Matrix-M1  adjuvant |  |  | rSARS-CoV-2 | Matrix-M1  adjuvant |  |
| A | 150-300 | | 0 | 0 | 0 | 0 |  | A | 0 | 0 |  |
| B | 150-300 | | 5 μg | 50 μg | 5 μg | 50 μg |  | B1  B | 0  5 μg | 0  50 μg |  |
| C | 150-300 | | 5 μg | 50 μg | 5 μg | 0 |  | C1  C2 | 0  5 μg | 0  50 μg |  |
| D | 150-300 | | 25 μg | 50 μg | 25 μg | 50 μg |  | D | 0 | 0 |  |
| E | 150-300 | | 25 μg | 50 μg | 25 μg | 0 |  | E | 0 | 0 |  |
| **B: Key Trial Timings** | | | | | | | | | | | |
| **Procedure** | | **Screening** | **Day** | | | | | | | | |
|  | | -45 to Day 0 | 0 | 7 | 21 | 28 | 35 | 105 | 189 | 217 | 357 |
| Vaccination | |  | **X** |  | **X** |  |  |  | **X** |  |  |
| Blood sample: immunogenicity (serology) | | X | **X** |  | **X** |  | **X** |  | **X** | **X** | **X** |
| Blood sample: immunogenicity (CMI subset) | |  |  | **X** |  | **X** |  |  |  |  |  |
| Nasal swab | | **X** |  | | | | | | | | |
| monitoring for COVID-19 disease endpoints | |  |  |  |  |  | | | | | |
| Reactogenicity – 7 days after each dose | |  |  | |  | |  |  |  |  |  |
| Unsolicited adverse event∞ | |  |  | | | | |  |  |  |  |
| Medically attended adverse event: All/related | |  |  | | | | | | | |  |
| Serious adverse event or adverse event of special interest | |  |  | | | | | | | | |

Abbreviations: CMI = cell-mediated immunity; SARS‑CoV-2 rS = severe acute respiratory syndrome coronavirus 2 recombinant spike protein nanoparticle vaccine.

| Table D. Overall Summary of Solicited and Unsolicited Adverse Events Following Vaccination of SARS-CoV-2 rS With Matrix-M1 Adjuvant in Adult Participants 18 to 84 Years of Age (Safety Analysis Set). | | | | | | |
| --- | --- | --- | --- | --- | --- | --- |
| **Vaccine Group** | **Group A** | **Group B** | **Group C** | **Group D** | **Group E** | **Total** |
| **Study Treatments Dose 1 / Dose 2NVX-CoV2373** | **Placebo / Placebo** | **5 µg + M1 /5 µg + M1** | **5 µg + M1 / Placebo** | **25 µg + M1 / 25 µg + M1** | **25 µg+ M1 / Placebo** |  |
| **Adverse Event Categories N** | **255** | **258** | **256** | **259** | **255** | **1283** |
| Any solicited local AE^*^ | 49 (19.2) | 190 (73.6) | 139 (54.3) | 217 (83.8) | 178 (69.8) | 773 (60.2) |
| Grade ≥3 solicited local AE | 0 | 13 (5.0) | 0 | 23 (8.9) | 1 (0.4) | 37 (2.9) |
| Any solicited systemic AE^*^ | 116 (45.5) | 160 (62.0) | 124 (48.4) | 179 (69.1) | 122 (47.8) | 701 (54.6) |
| Grade ≥3 solicited systemic AE | 6 (2.4) | 22 (8.5) | 6 (2.3) | 24 (9.3) | 3 (1.2) | 61 (4.8) |
| Any AEs through 35 days after first vaccination | 42 (16.5) | 51 (19.8) | 35 (13.7) | 52 (20.1) | 43 (16.9) | 223 (17.4) |
| Related AEs through 35 days after first vaccination | 6 (2.4) | 5 (1.9) | 2 (0.8) | 15 (5.8) | 3 (1.2) | 31 (2.4) |
| Severe AEs through 35 days after first vaccination | 3 (1.2) | 1 (0.4) | 4 (1.6) | 1 (0.4) | 0 | 9 (0.7) |
| Related severe AEs | 0 | 0 | 1 (0.4) | 0 | 0 | 1 (< 0.1) |
| Any AEs leading to vaccination discontinuation | 4 (1.6) | 1 (0.4) | 3 (1.2) | 4 (1.5) | 1 (0.4) | 13 (1.0) |
| Related AEs leading to vaccination discontinuation | 1 (0.4) | 0 | 1 (0.4) | 1 (0.4) | 0 | 3 (0.2) |
| Any AEs leading to study discontinuation | 2 (0.8) | 0 | 1 (0.4) | 4 (1.5) | 0 | 7 (0.5) |
| Related AEs leading to study discontinuation | 0 | 0 | 0 | 1 (0.4) | 0 | 1 (<0.1) |
| Any serious AEs | 2 (0.8) | 0 | 5 (2.0) | 1 (0.4) | 1 (0.4) | 9 (0.7) |
| Related serious AEs | 1 (0.4) | 0 | 1 (0.4) | 0 | 0 | 2 (0.2) |
| Any MAAEs | 14 (5.5) | 13 (5.0) | 20 (7.8) | 19 (7.3) | 18 (7.1) | 84 (6.5) |
| Related MAAEs | 2 (0.8) | 0 | 1 (0.4) | 2 (0.8) | 0 | 5 (0.4) |
| AESIs: PIMMC | 1 (0.4) | 0 | 0 | 0 | 0 | 1 (< 0.1) |
| Related AESIs: PIMMC | 1 (0.4) | 0 | 0 | 0 | 0 | 1 (< 0.1) |
| Any AESIs related to COVID-19 | 0 | 0 | 0 | 0 | 0 | 0 |

Abbreviations: AE = adverse event; AESI = adverse event of special interest; COVID-19 = coronavirus disease 2019; FDA = US Food and Drug Administration; MAAE = medically attended adverse event; PIMMC = potential immune-mediated medical conditions; SARS‑CoV-2 rS = severe acute respiratory syndrome coronavirus 2 recombinant spike protein nanoparticle vaccine.

*Includes solicited AEs reported by participants (via diary or spontaneously) with a recorded start date within 7 days post each vaccination window.

Note: Data are presented as number and percentage (n [%]) of participants. CIs not calculated.

### Table E. Percentage of All Participants (18 to 84 years) Experiencing Solicited Local and Systemic Adverse Events by Symptom, Vaccination Dose, Vaccine Group, and Maximum Toxicity Grade (Safety Analysis Set).*

| **Symptom** | **Vaccination Dose** | **Vaccine Group†** | | **N** | **Any Grade**  **n, % (95% CI)** | **Grade 1 (mild)**  **n, %** | **Grade 2 (moderate)**  **n, %** | **Grade 3 (severe)**  **n, %** | **Grade 4**  **(potentially life-threatening)**  **n, %** |
| --- | --- | --- | --- | --- | --- | --- | --- | --- | --- |
| Any solicited local AE | 1 | A  B  C  D  E  B+C  D+E | 0/0  5/50  5/50  25/50  25/50  5/50  25/50 | 252  253  255  252  253  508  505 | 39, 15.5 (11.2, 20.5)  131, 51.8 (45.4, 58.1)  135, 52.9 (46.6, 59.2)  154, 61.1 (54.8, 67.2)  175, 69.2 (63.1, 74.8)  266, 52.4 (47.9, 56.8)  329, 65.1 (60.8, 69.3) | 36, 14.3  94, 37.2  94, 36.9  99, 39.3  110, 43.5  188, 37.0  209, 41.4 | 3, 1.2  36, 14.2  41, 16.1  53, 21.0  64, 25.3  77, 15.2  69, 23.2 | 0, 0  1, 0.4  0, 0  2, 0.8  1, 0.4  1, 0.2  3, 0.6 | 0, 0  0, 0  0, 0  0, 0  0, 0  0, 0  0, 0 |
|  | 2 | A  B  C  D  E | 0/0  5/50  0/0  25/50  0/0 | 242  250  249  247  236 | 22, 9.1 (5.8, 13.4)  175, 70.0 (63.9, 75.6)  27, 10.8 (7.3, 15.4)  200, 81.0 (75.5, 85.7)  20, 8.5 (5.3, 12.8) | 20, 8.3  97, 38.8  20, 8.0  95, 38.5  18, 7.6 | 2, 0.8  65, 26.0  7, 2.8  84, 34.0  2, 0.8 | 0, 0  13, 5.2  0, 0  19, 7.7  0, 0 | 0, 0  0, 0  0, 0  2, 0.8  0, 0 |
| Pain | 1 | A  B  C  D  E  B+C  D+E | 0/0  5/50  5/50  25/50  25/50  5/50  25/50 | 252  253  255  252  253  508  505 | 10, 4.0 (1.9, 7.2)  68, 26.9 (21.5, 32.8)  71, 27.8 (22.4, 33.8)  83, 32.9 (27.2, 39.1)  105, 41.5 (35.4, 47.8)  139, 27.4 (23.5, 31.5)  188, 37.2 (33.0, 41.6) | 10, 4.0  65, 25.7  70, 27.5  81, 32.1  97, 38.3  135, 26.6  178, 35.2 | 0, 0  3, 1.2  1, 0.4  2, 0.8  8, 3.2  4, 0.8  10, 2.0 | 0, 0  0, 0  0, 0  0, 0  0, 0  0, 0  0, 0 | 0, 0  0, 0  0, 0  0, 0  0, 0  0, 0  0, 0 |
|  | 2 | A  B  C  D  E | 0/0  5/50  0/0  25/50  0/0 | 242  250  249  247  236 | 9, 3.7 (1.7, 6.9)  114, 45.6 (39.3, 52.0)  16, 6.4 (3.7, 10.2)  135, 54.7 (48.2, 61.0)  12, 5.1 (2.7, 8.7) | 9, 3.7  99, 39.6  15, 6.0  106, 42.9  12, 5.1 | 0, 0  10, 4.0  1, 0.4  23, 9.3  0, 0 | 0, 0  5, 2.0  0, 0  5, 2.0  0, 0 | 0, 0  0, 0  0, 0  1, 0.4  0, 0 |
| Erythema | 1 | A  B  C  D  E  B+C  D+E | 0/0  5/50  5/50  25/50  25/50  5/50  25/50 | 252  253  255  252  253  508  505 | 0, 0 (0.0, 1.5)  2, 0.8 (0.1, 2.8)  1, 0.4 (0.0, 2.2)  1, 0.4 (0.0, 2.2)  2, 0.8 (0.1, 2.8)  3, 0.6 (0.1, 1.7)  3, 0.6 (0.1, 1.7) | 0, 0  2, 0.8  1, 0.4  0, 0  0, 0  3, 0.6  0, 0 | 0, 0  0, 0  0, 0  1, 0.4  2, 0.8  0, 0  3, 0.6 | 0, 0  0, 0  0, 0  0, 0  0, 0  0, 0  0, 0 | 0, 0  0, 0  0, 0  0, 0  0, 0  0, 0  0, 0 |
|  | 2 | A  B  C  D  E | 0/0  5/50  0/0  25/50  0/0 | 242  250  249  247  236 | 0, 0 (0.0, 1.5)  12, 4.8 (2.5, 8.2)  0, 0 (0.0, 1.5)  33, 13.4 (9.4, 18.2)  0, 0 (0.0, 1.6) | 0, 0  4, 1.6  0, 0  7, 2.8  0, 0 | 0, 0  5, 2.0  0, 0  18, 7.3  0, 0 | 0, 0  3, 1.2  0, 0  8, 3.2  0, 0 | 0, 0  0, 0  0, 0  0, 0  0, 0 |
| Swelling | 1 | A  B  C  D  E  B+C  D+E | 0/0  5/50  5/50  25/50  25/50  5/50  25/50 | 252  253  255  252  253  508  505 | 0, 0 (0.0, 1.5)  2, 0.8 (0.1, 2.8)  3, 1.2 (0.2, 3.4)  1, 0.4 (0.0, 2.2)  3, 1.2 (0.2, 3.4)  5, 1.0 (0.3, 2.3)  4, 0.8 (0.2, 2.0) | 1, 0.4  2, 0.8  0, 0  1, 0.4  1, 0.4  2, 0.4  2, 0.4 | 0, 0  0, 0  3, 1.2  0, 0  2, 0.8  3, 0.6  2, 0.4 | 0, 0  0, 0  0, 0  0, 0  0, 0  0, 0  0, 0 | 0, 0  0, 0  0, 0  0, 0  0, 0  0, 0  0, 0 |
|  | 2 | A  B  C  D  E | 0/0  5/50  0/0  25/50  0/0 | 242  250  249  247  236 | 0, 0 (0.0, 1.5)  14, 5.6 (3.1, 9.2)  0, 0 (0.0, 1.5)  27, 10.9 (7.3, 15.5)  0, 0 (0.0, 1.6) | 0, 0  7, 2.8  0, 0  10, 4.0  0, 0 | 0, 0  6, 2.4  0, 0  12, 4.9  0, 0 | 0, 0  1, 0.4  0, 0  5, 2.0  0, 0 | 0, 0  0, 0  0, 0  0, 0  0, 0 |
| Tenderness | 1 | A  B  C  D  E  B+C  D+E | 0/0  5/50  5/50  25/50  25/50  5/50  25/50 | 252  253  255  252  253  508  505 | 33, 13.1 (9.2, 17.9)  122, 48.2 (41.9, 54.6)  122, 47.8 (41.6, 54.2)  142, 56.3 (50.0, 62.6)  158, 62.5 (56.2, 68.4)  244, 48.0 (43.6, 52.5)  300, 59.4 (55.0, 63.7) | 30, 11.9  86, 34.0  83, 32.5  89, 35.3  99, 39.1  169, 33.3  188, 37.2 | 3, 1.2  35, 13.8  39, 15.3  51, 20.2  58, 22.9  74, 14.6  109, 21.6 | 0, 0  1, 0.4  0, 0  2, 0.8  1, 0.4  1, 0.2  3, 0.6 | 0, 0  0, 0  0, 0  0, 0  0, 0  0, 0  0, 0 |
|  | 2 | A  B  C  D  E | 0/0  5/50  0/0  25/50  0/0 | 242  250  249  247  236 | 18, 7.4 (4.5, 11.5)  163, 65.2 (58.9, 71.1)  22, 13.1 (5.6, 13.1)  188, 76.1 (70.3, 81.3)  17, 7.2 (4.3, 11.3) | 16, 6.6  91, 36.4  16, 6.4  102, 41.3  15, 6.4 | 2, 0.8  63, 25.2  6, 2.4  73, 29.6  2, 0.8 | 0, 0  9, 3.6  0, 0  11, 4.5  0, 0 | 0, 0  0, 0  0, 0  2, 0.8  0, 0 |
| Any solicited systemic AE | 1 | A  B  C  D  E  B+C  D+E | 0/0  5/50  5/50  25/50  25/50  5/50  25/50 | 251  255  255  252  253  510  505 | 91, 36.3 (30.3, 42.5)  112, 43.9 (37.7, 50.2)  102, 40.0 (33.9, 46.3)  112, 44.4 (38.2, 50.8)  100, 39.5 (33.5, 45.8)  214, 42.0 (37.6, 46.4)  212, 42.0 (37.6, 46.4) | 52, 20.7  65, 25.5  60, 23.5  76, 30.2  60, 23.7  125, 24.5  136, 26.9 | 35, 13.9  37, 14.5  39, 15.3  33, 13.1  37, 14.6  76, 14.9  70, 13.9 | 2, 0.8  10, 3.9  3, 1.2  3, 1.2  3, 1.2  13, 2.5  6, 1.2 | 2, 0.8  0, 0  0, 0  0, 0  0, 0  0, 0  0, 0 |
|  | 2 | A  B  C  D  E | 0/0  5/50  0/0  25/50  0/0 | 241  250  249  247  235 | 66, 27.4 (21.9, 33.5)  132, 52.8 (46.4, 59.1)  71, 28.5 (23.0, 34.6)  157, 63.6 (57.2, 69.6)  51, 21.7 (16.6, 27.5) | 38, 15.8  56, 22.4  43, 17.3  60, 24.3  32, 13.6 | 25, 10.4  62, 24.8  24, 9.6  76, 30.8  19, 8.1 | 2, 0.8  14, 5.6  4, 1.6  20, 8.1  0, 0 | 0.4  0, 0  0, 0  0.4  0, 0 |
| Joint pain/ Arthralgia | 1 | A  B  C  D  E  B+C  D+E | 0/0  5/50  5/50  25/50  25/50  5/50  25/50 | 251  255  255  252  253  510  505 | 15, 6.0 (3.4, 9.7)  17, 6.7 (3.9, 10.5)  21, 8.2 (5.2, 12.3)  12, 4.8 (2.5, 8.2)  16, 6.3 (3.7, 10.1)  38, 7.5 (5.3, 10.1)  28, 5.5 (3.7, 7.9) | 11, 4.4  8, 3.1  11, 4.3  10, 4.0  8, 3.2  19, 3.7  18, 3.6 | 4, 1.6  7, 2.7  10, 3.9  2, 0.8  7, 2.8  17, 3.3  9, 1.8 | 0, 0  2, 0.8  0, 0  0, 0  1, 0.4  2, 0.4  1, 0.2 | 0, 0  0, 0  0, 0  0, 0  0, 0  0, 0  0, 0 |
|  | 2 | A  B  C  D  E | 0/0  5/50  0/0  25/50  0/0 | 241  250  249  247  235 | 9, 3.7 (1.7, 7.0)  37, 14.8 (10.6, 19.8)  8, 3.2 (1.4, 6.2)  47, 19.0 (14.3, 24.5)  4, 1.7 (0.5, 4.3) | 5, 2.1  18, 7.2  4, 1.6  15, 6.1  2, 0.9 | 4, 1.7  16, 6.4  4, 1.6  28, 11.3  2, 0.9 | 0, 0  3, 1.2  0, 0  4, 1.6  0, 0 | 0, 0, 0  0, 0, 0  0, 0  0, 0  0, 0 |
| Fatigue | 1 | A  B  C  D  E  B+C  D+E | 0/0  5/50  5/50  25/50  25/50  5/50  25/50 | 251  255  255  252  253  510  505 | 52, 20.7 (15.9, 26.3)  59, 23.1 (18.1, 28.8)  62, 24.3 (19.2, 30.1)  41, 16.3 (11.9, 21.4)  47, 18.6 (14.0, 23.9)  121, 23.7 (20.1, 27.7)  88, 17.4 (14.2, 21.0) | 27, 10.8  29, 11.4  34, 13.3  23, 9.1  28, 11.1  63, 12.4  51, 10.1 | 24, 9.6  25, 9.8  25, 9.8  16, 6.3  18, 7.1  50, 9.8  34, 6.7 | 1, 0.4  5, 2.0  3, 1.2  2, 0.8  1, 0.4  8, 1.6  3, 0.6 | 0, 0  0, 0  0, 0  0, 0  0, 0  0, 0  0, 0 |
|  | 2 | A  B  C  D  E | 0/0  5/50  0/0  25/50  0/0 | 241  250  249  247  235 | 33, 13.7 (9.6, 18.7)  89, 35.6 (29.7, 41.9)  44, 17.7 (13.1, 23.0)  105, 42.5 (36.3, 48.9)  31, 13.2 (9.1, 18.2) | 16, 6.6  32, 12.8  24, 9.6  31, 12.6  18, 7.7 | 16, 6.6  50, 20.0  18, 7.2  58, 23.5  13, 5.5 | 1, 0.4  7, 2.8  2, 0.8  16, 6.5  0, 0 | 0, 0  0, 0  0, 0  0, 0  0, 0 |
| Malaise | 1 | A  B  C  D  E  B+C  D+E | 0/0  5/50  5/50  25/50  25/50  5/50  25/50 | 251  255  255  252  253  510  505 | 30, 12.0 (8.2, 16.6)  31, 12.2 (8.4, 16.8)  31, 12.2 (8.4, 16.8)  23, 9.1 (5.9, 13.4)  26, 10.3 (6.8, 14.7)  62, 12.2 (9.4, 15.3)  49, 9.7 (7.3, 12.6) | 16, 6.4  16 , 6.3  17, 6.7  14, 5.6  18, 7.1  33, 6.5  32, 6.3 | 13, 5.2  9, 3.5  12, 4.7  7, 2.8  8, 3.2  21, 4.1  15, 3.0 | 0, 0  6, 2.4  2, 0.8  2, 0.8  0, 0  8, 1.6  2, 0.4 | 1, 0.4  0, 0  0, 0  0, 0  0, 0  0, 0  0, 0 |
|  | 2 | A  B  C  D  E | 0/0  5/50  0/0  25/50  0/0 | 241  250  249  247  235 | 19, 17.9 (4.8, 12.0)  66, 26.4 (21.0, 32.3)  19, 7.6 (4.7, 11.7)  74, 30.0 (24.3, 36.1)  11, 4.7 (2.4, 8.2) | 12, 5.0  23, 9.2  10, 4.0  19, 7.7  9, 3.8 | 7, 2.9  37, 14.8  8, 3.2  44, 17.8  2, 0.9 | 0, 0  6, 2.4  1, 0.4  11, 4.5  0, 0 | 0, 0  0, 0  0, 0  0, 0  0, 0 |
| Temperature/ Fever | 1 | A  B  C  D  E  B+C  D+E | 0/0  5/50  5/50  25/50  25/50  5/50  25/50 | 248  255  255  252  253  510  505 | 6, 2.4 (0.9, 5.2)  6, 2.4 (0.9, 5.1)  6, 2.4 (0.9, 5.1)  3, 1.2 (0.2, 3.4)  3, 1.2 (0.2, 3.4)  12, 2.4 (1.2, 4.1)  6, 1.2 (0.4, 2.6) | 2, 0.8  3, 1.2  4, 1.6  0, 0  1, 0.4  7, 1.4  1, 0.2 | 3, 1.2  0, 0  2, 0.8  2, 0.8  2, 0.8  2, 0.4  4, 0.8 | 0, 0  3, 1.2  0, 0  1, 0.4  0, 0  3, 0.6  1, 0.2 | 1, 0.4  0, 0  0, 0  0, 0  0, 0  0, 0  0, 0 |
|  | 2 | A  B  C  D  E | 0/0  5/50  0/0  25/50  0/0 | 239  249  249  245  230 | 2, 0.8 (0.1, 3.0)  11, 4.4 (2.2, 7.8)  1, 0.4 (0.0, 2.2)  20, 8.2 (5.1, 12.3)  1, 0.4 (0.0, 2.4) | 1, 0.4  8, 3.2  0, 0  9, 3.7  0, 0 | 0, 0  2, 0.8  1, 0.4  9, 3.7  1, 0.4 | 0, 0  1, 0.4  0, 0  2, 0.8  0, 0 | 1, 0.4  0, 0  0, 0  0, 0  0, 0 |
| Headache | 1 | A  B  C  D  E  B+C  D+E | 0/0  5/50  5/50  25/50  25/50  5/50  25/50 | 251  255  255  252  253  510  505 | 48, 19.1 (14.4, 24.5)  55, 21.6 (16.7, 27.1)  42, 16.5 (12.1, 21.6)  48, 19.0 (14.4, 24.4)  38, 15.0 (10.9, 20.0)  97, 19.0 (15.7, 22.7)  86, 17.0 (13.9, 20.6) | 38, 15.1  40, 15.7  33, 12.9  41, 16.3  29, 11.5  73, 14.3  70, 13.9 | 9, 3.6  14, 5.5  9, 3.5  7, 2.8  8, 3.2  23, 4.5  15, 3.0 | 1, 0.4  1, 0.4  0, 0  0, 0  1, 0.4  1, 0.2  1, 0.2 | 0, 0  0, 0  0, 0  0, 0  0, 0  0, 0  0, 0 |
|  | 2 | A  B  C  D  E | 0/0  5/50  0/0  25/50  0/0 | 241  250  249  247  235 | 31, 12.9 (8.9, 17.8)  74, 29.6 (24.0, 35.7)  32, 12.9 (9.0, 17.7)  84, 34.0 (28.1, 40.3)  28, 11.9 (8.1, 16.8) | 25, 10.4  42, 16.8  26, 10.4  41, 16.6  24, 10.2 | 5, 2.1  27, 10.8  5, 2.0  37, 15.0  4, 1.7 | 1, 0.4  5, 2.0  1, 0.4  6, 2.4  0, 0 | 0, 0  0, 0  0, 0  0, 0  0, 0 |
| Muscle pain/ Myalgia | 1 | A  B  C  D  E  B+C  D+E | 0/0  5/50  5/50  25/50  25/50  5/50  25/50 | 251  255  255  252  253  510  505 | 27, 10.8 (7.2, 15.3)  51, 20.0 (15.3, 25.4)  52, 20.4 (15.6, 25.9)  59, 23.4 (18.3, 29.1)  48, 19.0 (14.3, 24.4)  103, 20.2 (16.8, 23.9)  107, 21.2 (17.7, 25.0) | 20, 8.0  36, 14.1  40, 15.7  45, 17.9  38, 15.0  76, 14.9  83, 16.4 | 7, 2.8  13, 5.1  12, 4.7  14, 5.6  8, 3.2  25, 4.9  22, 4.4 | 0, 0  2, 0.8  0, 0  0, 0  2, 0.8  2, 0.4  2, 0.4 | 0, 0  0, 0  0, 0  0, 0  0, 0  0, 0  0, 0 |
|  | 2 | A  B  C  D  E | 0/0  5/50  0/0  25/50  0/0 | 241  250  249  247  235 | 16, 6.6 (3.8, 10.6)  77, 30.8 (25.1, 36.9)  18, 7.2 (4.3, 11.2)  101, 40.9 (34.7, 47.3)  5, 2.1 (0.7, 4.9) | 11, 4.6  34, 13.6  12, 4.8  46, 18.6  3, 1.3 | 5, 2.1  37, 14.8  5, 2.0  46, 18.6  2, 0.9 | 0, 0  6, 2.4  1, 0.4  8, 3.2  0, 0 | 0,0  0,0  0,0  1, 0.4  0,0 |
| Nausea or vomiting | 1 | A  B  C  D  E  B+C  D+E | 0/0  5/50  5/50  25/50  25/50  5/50  25/50 | 251  255  255  252  253  510  505 | 9, 3.6 (1.7, 6.7)  15, 5.9 (3.3, 9.5)  10, 3.9 (1.9, 7.1)  11, 4.4 (2.2, 7.7)  16, 6.3 (3.7, 10.1)  25, 4.9 (3.2, 7.2)  27, 5.3 (3.6, 7.7) | 8, 3.2  11, 4.3  8, 3.1  10, 4.0  15, 5.9  19, 3.7  25, 5.0 | 1, 0.4  3, 1.2  2, 0.8  1, 0.4  1, 0.4  5, 1.0  2, 0.4 | 0, 0  1, 0.4  0, 0  0, 0  0, 0  1, 0.2  0, 0 | 0, 0  0, 0  0, 0  0, 0  0, 0  0, 0  0, 0 |
|  | 2 | A  B  C  D  E | 0/0  5/50  0/0  25/50  0/0 | 241  250  249  247  235 | 9, 3.7 (1.7, 7.0)  18, 7.2 (4.3, 11.1)  5, 2.0 (0.7, 4.6)  27, 10.9 (7.3, 15.5)  6, 2.6 (0.9, 5.5) | 9, 3.7  15, 6.0  4, 1.6  15, 6.1  5, 2.1 | 0, 0  3, 1.2  1, 0.4  11, 4.5  1, 0.4 | 0, 0  0, 0  0, 0  1, 0.4  0, 0 | 0, 0  0, 0  0, 0  0, 0  0, 0 |

Abbreviations: AE = adverse event; CI = confidence interval; SARS‑CoV-2 rS = severe acute respiratory syndrome coronavirus 2 recombinant spike protein nanoparticle vaccine.

* N denotes number of participants assessed.

† Group A (placebo): 0 µg rSARS-CoV-2/0 µg Matrix-M1 adjuvant on first vaccination; 0 µg rSARS-CoV-2/0 µg Matrix-M1 adjuvant on second vaccination. Group B: 5 µg rSARS-CoV-2/50 µg Matrix-M1 adjuvant on first vaccination; 5 µg rSARS-CoV-2/50 µg Matrix-M1 adjuvant on second vaccination. Group C: 5 µg rSARS-CoV-2/50 µg Matrix-M1 adjuvant on first vaccination; 0 µg rSARS-CoV-2/0 µg Matrix-M1 adjuvant on second vaccination. Group D: 25 µg rSARS-CoV-2/50 µg Matrix-M1 adjuvant on first vaccination; 25 µg rSARS-CoV-2/50 µg Matrix-M1 adjuvant on second vaccination. Group E: 25 µg rSARS-CoV-2/50 µg Matrix-M1 adjuvant on first vaccination; 0 µg rSARS-CoV-2/0 µg Matrix-M1 adjuvant on second vaccination.

### Table F. Percentage of Younger Adults (18 to 59 Years) Experiencing Solicited Local and Systemic Adverse Events by Symptom, Vaccination Dose, Vaccine Group, and Maximum Toxicity Grade (Safety Analysis Set).*

| **Symptom** | **Vaccination Dose** | **Vaccine Group†** | | **N** | **Any Grade**  **n, % (95% CI)** | **Grade 1 (mild)**  **n, %** | **Grade 2 (moderate)**  **n, %** | **Grade 3 (severe)**  **n, %** | **Grade 4**  **(potentially life-threatening)**  **n, %** |
| --- | --- | --- | --- | --- | --- | --- | --- | --- | --- |
| Any solicited local AE | 1 | A  B  C  D  E  B+C  D+E | 0/0  5/50  5/50  25/50  25/50  5/50  25/50 | 138  139  138  139  136  277  275 | 25, 18.1 (12.1, 25.6)  87, 62.6 (54.0, 70.6)  92, 66.7 (58.1, 74.5)  104, 74.8 (66.8, 81.8)  105, 77.2 (69.2, 84.0)  179, 64.6 (58.7, 70.2)  209, 76.0 (70.5, 80.9) | 23, 16.7  53, 38.1  56, 40.6  56, 40.3  59, 43.4  109, 39.4  115, 41.8 | 2, 1.4  33, 23.7  36, 26.1  46, 33.1  46, 33.8  71, 24.9  92, 33.5 | 0, 0  1, 0.7  0, 0  2, 1.4  0, 0  1, 0.4  2, 0.7 | 0, 0  0, 0  0, 0  0, 0  0, 0  0, 0  0, 0 |
|  | 2 | A  B  C  D  E | 0/0  5/50  0/0  25/50  0/0 | 132  137  136  137  127 | 15, 11.4 (6.5, 18.0)  105, 76.6 (68.7, 83.4)  19, 14.0 (8.6, 21.0)  126, 92.0 (86.1, 95.9)  9, 7.1 (3.3, 13.0) | 14, 10.6  49, 35.8  14, 10.3  52, 38.0  5.5 | 1, 0.8  46, 33.6  5, 3.7  63, 46.0  1.6 | 0, 0  10, 7.3  0, 0  10, 7.3  0, 0 | 0, 0  0, 0  0, 0  1, 0.7  0, 0 |
| Pain | 1 | A  B  C  D  E  B+C  D+E | 0/0  5/50  5/50  25/50  25/50  5/50  25/50 | 138  139  138  139  136  277  275 | 8, 5.8 (2.5, 11.1)  48, 34.5 (26.7, 43.1)  51, 37.0 (28.9, 45.6)  62, 44.6 (36.2, 53.3)  69, 50.7 (42.0, 59.4)  99, 35.7 (30.1, 41.7)  131, 47.6 (41.6, 53.7) | 8, 5.8  46, 33.1  50, 36.2  60, 43.2  61, 44.9  96, 34.7  121, 44.0 | 0, 0  2, 1.4  1, 0.7  2, 1.4  8, 5.9  3, 1.0  10, 3.6 | 0, 0  0, 0  0, 0  0, 0  0, 0  0, 0  0, 0 | 0, 0  0, 0  0, 0  0, 0  0, 0  0, 0  0, 0 |
|  | 2 | A  B  C  D  E | 0/0  5/50  0/0  25/50  0/0 | 132  137  136  137  127 | 6, 4.5 (1.7, 9.6)  68, 49.6 (41.0, 58.3)  11, 8.1 (4.1, 14.0)  92, 67.2 (58.6, 74.9)  6, 4.7 (1.8, 10.0) | 6, 4.5  54, 39.4  10, 7.4  72, 52.6  6, 4.7 | 0, 0  10, 7.3  1, 0.7  15, 10.9  0, 0 | 0, 0  4, 2.9  0, 0  5, 3.6  0, 0 | 0, 0  0, 0  0, 0  0, 0  0, 0 |
| Erythema | 1 | A  B  C  D  E  B+C  D+E | 0/0  5/50  5/50  25/50  25/50  5/50  25/50 | 138  139  138  139  136  277  275 | 0, 0 (0.0, 2.6)  2, 1.4 (0.2, 5.1)  0, 0 (0.0, 2.6)  0, 0 (0.0, 2.6)  1, 0.7 (0.0, 4.0)  2, 0.7 (0.1, 2.6)  1, 0.4 (0.0, 2.0) | 0, 0  2, 1.4  0, 0  0, 0  0, 0  2, 0.7  0, 0 | 0, 0  0, 0  0, 0  0, 0  1, 0.7  0, 0  1, 0.4 | 0, 0  0, 0  0, 0  0, 0  0, 0  0, 0  0, 0 | 0, 0  0, 0  0, 0  0, 0  0, 0  0, 0  0, 0 |
|  | 2 | A  B  C  D  E | 0/0  5/50  0/0  25/50  0/0 | 132  137  136  137  127 | 0, 0 (0.0, 2.8)  6, 4.4 (1.6, 9.3)  0, 0 (0.0, 2.7)  16, 11.7 (6.8, 18.3)  0, 0 (0.0, 2.9) | 0, 0  2, 2.2  0, 0  4, 2.9  0, 0 | 0, 0  2, 0.7  0, 0  10, 7.3  0, 0 | 0, 0  2, 0.7  0, 0  2, 1.5  0, 0 | 0, 0  0, 0  0, 0  0, 0  0, 0 |
| Swelling | 1 | A  B  C  D  E  B+C  D+E | 0/0  5/50  5/50  25/50  25/50  5/50  25/50 | 138  139  138  139  136  277  275 | 1, 0.7 (0.0, 4.0)  2, 1.4 (0.2, 5.1)  2, 1.4 (0.2, 5.1)  1, 0.7 (0.0, 3.9)  0, 0 (0.0, 2.7)  4, 1.4 (0.4, 3.7)  1, 0.4 (0.0, 2.0) | 1, 0.7  2, 1.4  0, 0  1, 0.7  0, 0  2, 0.7  1, 0.4 | 0, 0  0, 0  2, 1.4  0, 0  0, 0  2, 0.7  0, 0 | 0, 0  0, 0  0, 0  0, 0  0, 0  0, 0  0, 0 | 0, 0  0, 0  0, 0  0, 0  0, 0  0, 0  0, 0 |
|  | 2 | A  B  C  D  E | 0/0  5/50  0/0  25/50  0/0 | 132  137  136  137  127 | 0, 0 (0.0, 2.8)  5, 3.6 (1.2, 8.3)  0, 0 (0.0, 2.7)  16, 11.7 (6.8, 18.3)  0, 0 (0.0, 2.9) | 0, 0  3, 2.2  0, 0  7, 5.1  0, 0 | 0, 0  1, 0.7  0, 0  7, 5.1  0, 0 | 0, 0  1, 0.7  0, 0  2, 1.5  0, 0 | 0, 0  0, 0  0, 0  0, 0  0, 0 |
| Tenderness | 1 | A  B  C  D  E  B+C  D+E | 0/0  5/50  5/50  25/50  25/50  5/50  25/50 | 138  139  138  139  136  277  275 | 21, 15.2 (9.7, 22.3)  83, 59.7 (51.1, 67.9)  85, 61.6 (52.9, 69.7)  96, 69.1 (60.7, 76.6)  92, 67.6 59.1, 75.4)  168, 60.6 (54.6, 66.4)  188, 68.4 (62.5, 73.8) | 19, 13.8  50, 36.0  51, 37.0  49, 35.3  50, 36.8  101, 36.5  99, 36.0 | 2, 1.4  32, 23.0  34, 24.6  45, 32.4  42, 30.9  66, 23.8  87, 31.6 | 0, 0  1, 0.7  0, 0  2, 1.4  0, 0  1, 0.4  2, 0.7 | 0, 0  0, 0  0, 0  0, 0  0, 0  0, 0  0, 0 |
|  | 2 | A  B  C  D  E | 0/0  5/50  0/0  25/50  0/0 | 132  137  136  137  127 | 12, 9.1 (4.8, 15.3)  101, 73.7 (65.5, 80.9)  17, 12.5 (7.5, 19.3)  120, 87.6 (80.9, 92.6)  8, 6.3 (2.8, 12.0) | 11, 8.3  48, 35.0  13, 9.6  56, 40.9  6, 4.7 | 1, 0.8  45, 32.8  4, 2.9  55, 40.1  2, 1.6 | 0, 0  8, 5.8  0, 0  8, 5.8  0, 0 | 0, 0  0, 0  0, 0  1, 0.7  0, 0 |
| Any solicited systemic AE | 1 | A  B  C  D  E  B+C  D+E | 0/0  5/50  5/50  25/50  25/50  5/50  25/50 | 138  139  138  139  136  277  275 | 55, 39.9 (31.6, 48.5)  68, 48.9 (40.4, 57.5)  66, 47.8 (39.3, 56.5)  79, 56.8 (48.2, 65.2)  60, 44.1 (35.6, 52.9)  134, 48.4 (42.4, 54.4)  139, 50.5 (44.5, 56.6) | 29, 21.0  39, 28.1  41, 29.7  52, 37.4  32, 23.5  80, 28.9  82, 30.5 | 23, 16.7  23, 16.5  24, 17.4  26, 18.7  25, 18.4  47, 17.0  51, 18.5 | 2, 1.4  6, 4.3  1, 0.7  1, 0.7  3, 2.2  7, 2.5  4, 1.5 | 1, 0.7  0, 0  0, 0  0, 0  0, 0  0, 0  0, 0 |
|  | 2 | A  B  C  D  E | 0/0  5/50  0/0  25/50  0/0 | 132  137  136  137  127 | 42, 31.8 (24.0, 40.5)  88, 64.2 (55.6, 72.2)  46, 33.8 (25.9, 42.4)  101, 73.7 (65.5, 80.9)  29, 22.8 (15.9, 31.1) | 22, 16.7  34, 24.8  28, 20.6  37, 27.0  18, 14.2 | 18, 13.6  43, 31.4  16, 11.8  48, 35.0  11, 8.7 | 1, 0.8  11, 8.0  2, 1.5  16, 11.7  0, 0 | 1, 0.8  0, 0  0, 0  0, 0  0, 0 |
| Joint pain/ Arthralgia | 1 | A  B  C  D  E  B+C  D+E | 0/0  5/50  5/50  25/50  25/50  5/50  25/50 | 138  139  138  139  136  277  275 | 12, 8.7 (4.6, 14.7)  11, 7.9 (4.0, 13.7)  8, 5.8 (2.5, 11.1)  8, 5.8 (2.5, 11.0)  6, 4.4 (1.6, 9.4)  19, 6.9 (4.2, 10.5)  14, 5.1 (2.8, 8.4) | 8, 5.8  7, 5.0  4, 2.9  6, 4.3  3, 2.2  11, 4.0  9, 3.3 | 4, 2.9  3, 2.2  4, 2.9  2, 1.4  2, 1.5  7, 2.5  4, 1.5 | 0, 0  1, 0.7  0, 0  0, 0  1, 0.7  1, 0.4  1, 0.4 | 0, 0  0, 0  0, 0  0, 0  0, 0  0, 0  0, 0 |
|  | 2 | A  B  C  D  E | 0/0  5/50  0/0  25/50  0/0 | 132  137  136  137  127 | 7, 5.3 (2.2, 10.6)  25, 18.2 (12.2, 25.7)  4, 2.9 (0.8, 7.4)  33, 24.1 (17.2, 32.1)  0, 0 (0.0, 2.9) | 5, 3.8  12, 8.8  3, 2.2  10, 7.3  0, 0 | 2, 1.5  12, 8.8  1, 0.7  19, 13.9  0, 0 | 0, 0  1, 0.7  0, 0  4, 2.9  0, 0 | 0, 0  0, 0  0, 0  0, 0  0, 0 |
| Fatigue | 1 | A  B  C  D  E  B+C  D+E | 0/0  5/50  5/50  25/50  25/50  5/50  25/50 | 138  139  138  139  136  277  275 | 32, 23.2 (16.4, 31.1)  37, 26.6 (19.5, 34.8)  41, 29.7 (22.2, 38.1)  29, 20.9 (14.4, 28.6)  31, 22.8 (16.0, 30.8)  78, 28.2 (22.9, 33.9)  60, 21.8 (17.1, 27.2) | 16, 11.6  20, 14.4  24, 17.4  18, 12.9  20, 14.7  44, 15.9  38, 13.8 | 15, 10.9  14, 10.1  16, 11.6  10, 7.2  10, 7.4  26, 10.8  20, 7.3 | 1, 0.7  3, 2.2  1, 0.7  1, 0.7  1, 0.7  4, 1.4  2, 0.7 | 0, 0  0, 0  0, 0  0, 0  0, 0  0, 0  0, 0 |
|  | 2 | A  B  C  D  E | 0/0  5/50  0/0  25/50  0/0 | 132  137  136  137  127 | 22, 16.7 (10.7, 24.1)  63, 46.0 (37.4, 54.7)  31, 22.8 (16.0, 30.8)  69, 50.4 (41.7, 59.0)  20, 15.7 (9.9, 23.3) | 10, 7.6  23, 16.8  17, 12.5  20, 14.6  14, 11.0 | 12, 9.1  33, 24.1  13, 9.6  36, 26.3  6, 4.7 | 0, 0  7, 5.1  1, 0.7  13, 9.5  0, 0 | 0, 0  0, 0  0, 0  0, 0  0, 0 |
| Malaise | 1 | A  B  C  D  E  B+C  D+E | 0/0  5/50  5/50  25/50  25/50  5/50  25/50 | 138  139  138  139  136  277  275 | 21, 15.2 (9.7, 22.3)  13, 19.4 (5.1, 15.5)  18, 13.0 (7.9, 19.8)  18, 12.9 (7.9, 19.7)  14, 10.3 (5.7, 16.7)  31, 11.2 (7.7, 15.5)  32, 11.6 (8.1, 16.0) | 13, 9.4  6, 4.3  10, 7.2  11, 7.9  9, 6.6  16, 5.8  20, 7.3 | 8, 5.8  3, 2.2  8, 5.8  6, 4.3  5, 3.7  11, 4.0  11, 4.0 | 0, 0  4, 2.9  0, 0  1, 0.7  0, 0  4, 1.4  1, 0.4 | 0, 0  0, 0  0, 0  0, 0  0, 0  0, 0  0, 0 |
|  | 2 | A  B  C  D  E | 0/0  5/50  0/0  25/50  0/0 | 132  137  136  137  127 | 11, 8.3 (4.2, 14.4)  43, 31.4 (23.7, 39.9)  12, 8.8 (4.6, 14.9)  56, 40.9 (32.6, 49.6)  7, 5.5 (2.2, 11.0) | 7, 5.3  15, 10.9  7, 5.1  16, 11.7  6, 4.7 | 4, 3.0  23, 16.8  5, 3.7  31, 22.6  1, 0.8 | 0, 0  5, 3.6  0, 0  9, 6.6  0, 0 | 0, 0  0, 0  0, 0  0, 0  0, 0 |
| Temperature/ Fever | 1 | A  B  C  D  E  B+C  D+E | 0/0  5/50  5/50  25/50  25/50  5/50  25/50 | 136  139  138  139  136  277  275 | 3, 2.2 (0.5, 6.3)  2, 1.4 (0.2, 5.1)  3, 2.2 (0.5, 6.2)  1, 0.7 (0.0, 3.9)  2, 1.5 (0.2, 5.2)  5, 1.8 (0.6, 4.2)  3, 1.1 (0.2, 3.2) | 1, 0.7  1, 0.7  2, 1.4  0, 0  1, 0.7  3, 1.1  1, 0.4 | 1, 0.7  0, 0  1, 0.7  1, 0.7  1, 0.7  1, 0.4  2, 0.7 | 0, 0  1, 0.7  0, 0  0, 0  0, 0  1, 0.4  0, 0 | 1, 0.7  0, 0  0, 0  0, 0  0, 0  0, 0  0, 0 |
|  | 2 | A  B  C  D  E | 0/0  5/50  0/0  25/50  0/0 | 130  137  136  137  123 | 1, 0.8 (0.0, 4.2)  9, 6.6 (3.0, 12.1)  1, 0.7 (0.0, 4.0)  16, 11.7 (6.8, 18.3)  0, 0 (0.0, 3.0) | 0, 0  6, 4.4  0, 0  6, 4.4  0, 0 | 0, 0  2, 1.5  1, 0.7  8, 5.8  0, 0 | 0, 0  1, 0.7  0, 0  2, 1.5  0, 0 | 1, 0.8  0, 0  0, 0  0, 0  0, 0 |
| Headache | 1 | A  B  C  D  E  B+C  D+E | 0/0  5/50  5/50  25/50  25/50  5/50  25/50 | 138  139  138  139  136  277  275 | 31, 22.5 (15.8, 30.3)  36, 25.9 (18.8, 34.0)  30, 21.7 (15.2, 29.6)  35, 25.2 (18.2, 33.2)  26, 19.1 (12.9, 26.7)  66, 23.8 (18.9, 29.3)  61, 22.2 (17.4, 27.6) | 23, 16.7  25, 18.0  24, 17.4  29, 20.9  18, 13.2  49, 17.7  47, 17.1 | 7, 5.1  10, 7.2  6, 4.3  6, 4.3  7, 5.1  16, 5.8  13, 4.7 | 1, 0.7  1, 0.7  0, 0  0, 0  1, 0.7  1, 0.4  1, 0.4 | 0, 0  0, 0  0, 0  0, 0  0, 0  0, 0  0, 0 |
|  | 2 | A  B  C  D  E | 0/0  5/50  0/0  25/50  0/0 | 132  137  136  137  127 | 19, 14.4 (8.9, 21.6)  57, 41.6 (33.3, 50.3)  20, 14.7 (9.2, 21.8)  56, 40.9 (32.6, 49.6)  16, 12.6 (7.4, 19.7) | 14, 10.6  32, 23.4  17, 12.5  27, 19.7  13, 10.2 | 4, 3.0  21, 15.3  3, 2.2  24, 17.5  3, 2.4 | 1, 0.8  4, 2.9  0, 0  5, 3.6  0, 0 | 0, 0  0, 0  0, 0  0, 0  0, 0 |
| Muscle pain/ Myalgia | 1 | A  B  C  D  E  B+C  D+E | 0/0  5/50  5/50  25/50  25/50  5/50  25/50 | 138  139  138  139  136  277  275 | 18, 13.0 (7.9, 19.8)  35, 25.2 (18.2, 33.2)  33, 23.9 (17.1, 31.9)  47, 33.8 (26.0, 42.3)  27, 19.9 (13.5, 27.6)  68, 24.5 (19.6, 30.1)  74, 26.9 (21.8, 32.6) | 14, 10.1  24, 17.3  24, 17.4  34, 24.5  20, 14.7  48, 17.3  54, 19.6 | 4, 2.9  10, 7.2  9, 6.5  13, 9.4  5, 3.7  19, 6.9  18, 6.5 | 0, 0  1, 0.7  0, 0  0, 0  2, 1.5  1, 0.4  2, 0.7 | 0, 0  0, 0  0, 0  0, 0  0, 0  0, 0  0, 0 |
|  | 2 | A  B  C  D  E | 0/0  5/50  0/0  25/50  0/0 | 132  137  136  137  127 | 15, 11.4 (6.5, 18.0)  55, 40.1 (31.9, 48.9)  14, 10.3 (5.7, 16.7)  69, 50.4 (41.7, 59.0)  2, 1.6 (0.2, 5.6) | 11, 8.3  24, 17.5  10, 7.4  31, 22.6  2, 1.6 | 4, 3.0  26, 19.0  3, 2.2  30, 21.9  0, 0 | 0, 0  5, 3.6  1, 0.7  8, 5.8  0, 0 | 0, 0  0, 0  0, 0  0, 0  0, 0 |
| Nausea or vomiting | 1 | A  B  C  D  E  B+C  D+E | 0/0  5/50  5/50  25/50  25/50  5/50  25/50 | 138  139  138  139  136  277  275 | 7, 5.1 (2.1, 10.2)  9, 6.5 (3.0, 11.9)  6, 4.3 (1.6, 9.2)  8, 5.8 (2.5, 11.0)  13, 9.6 (5.2, 15.8)  15, 5.4 (3.1, 8.8)  21, 7.6 (4.8, 11.4) | 6, 4.3  8, 5.8  5, 3.6  7, 5.0  12, 8.8  13, 4.7  19, 6.9 | 1, 0.7  0, 0  1, 0.7  1, 0.7  1, 0.7  1, 0.4  2, 0.7 | 0, 0  1, 0.7  0, 0  0, 0  0, 0  1, 0.4  0, 0 | 0, 0  0, 0  0, 0  0, 0  0, 0  0, 0  0, 0 |
|  | 2 | A  B  C  D  E | 0/0  5/50  0/0  25/50  0/0 | 132  137  136  137  127 | 8, 6.1 (2.7, 11.6)  11, 8.0 (4.1, 13.9)  4, 2.9 (0.8, 7.4)  19, 13.9 (8.6, 20.8)  5, 3.9 (1.3, 8.9) | 8, 6.1  9, 6.6  4, 2.9  9, 6.6  4, 3.1 | 0, 0  2, 1.5  0, 0  9, 6.6  1, 0.8 | 0, 0  0, 0  0, 0  1, 0.7  0, 0 | 0, 0  0, 0  0, 0  0, 0  0, 0 |

Abbreviations: AE = adverse event; CI = confidence interval; SARS‑CoV-2 rS = severe acute respiratory syndrome coronavirus 2 recombinant spike protein nanoparticle vaccine.

* N denotes number of participants assessed.

† Group A (placebo): 0 µg rSARS-CoV-2/0 µg Matrix-M1 adjuvant on first vaccination; 0 µg rSARS-CoV-2/0 µg Matrix-M1 adjuvant on second vaccination. Group B: 5 µg rSARS-CoV-2/50 µg Matrix-M1 adjuvant on first vaccination; 5 µg rSARS-CoV-2/50 µg Matrix-M1 adjuvant on second vaccination. Group C: 5 µg rSARS-CoV-2/50 µg Matrix-M1 adjuvant on first vaccination; 0 µg rSARS-CoV-2/0 µg Matrix-M1 adjuvant on second vaccination. Group D: 25 µg rSARS-CoV-2/50 µg Matrix-M1 adjuvant on first vaccination; 25 µg rSARS-CoV-2/50 µg Matrix-M1 adjuvant on second vaccination. Group E: 25 µg rSARS-CoV-2/50 µg Matrix-M1 adjuvant on first vaccination; 0 µg rSARS-CoV-2/0 µg Matrix-M1 adjuvant on second vaccination.

### Table G. Percentage of Older Adults (60 to 84 Years) Experiencing Solicited Local and Systemic Adverse Events by Symptom, Vaccination Dose, Vaccine Group, and Maximum Toxicity Grade (Safety Analysis Set).*

| **Symptom** | **Vaccination Dose** | **Vaccine Group†** | | **N** | **Any Grade**  **n, % (95% CI)** | **Grade 1 (mild)**  **n, %** | **Grade 2 (moderate)**  **n, %** | **Grade 3 (severe)**  **n, %** | **Grade 4**  **(potentially life-threatening)**  **n, %** |
| --- | --- | --- | --- | --- | --- | --- | --- | --- | --- |
| Any solicited local AE | 1 | A  B  C  D  E  B+C  D+E | 0/0  5/50  5/50  25/50  25/50  5/50  25/50 | 114  114  117  113  117  231  230 | 14, 12.3 (6.9, 19.7)  44, 38.6 (29.6, 48.2)  43, 36.8 (28.0, 46.2)  50, 44.2 (34.9, 53.9)  70, 59.8 (50.4, 68.8)  87, 37.7 (31.4, 44.3)  120, 52.2 (45.5,58.8) | 13, 11.4  41, 36.0  38, 32.5  43, 38.1  51, 43.6  79, 34.2  94, 40.1 | 1, 0.9  3, 2.6  5, 4.3  7, 6.2  18, 15.4  8, 3.5  25, 10.9 | 0, 0  0, 0  0, 0  0, 0  1, 0.9  0, 0  1, 0.4 | 0, 0  0, 0  0, 0  0, 0  0, 0  0, 0  0, 0 |
|  | 2 | A  B  C  D  E | 0/0  5/50  0/0  25/50  0/0 | 110  113  113  110  109 | 7, 6.4 (2.6, 12.7)  70, 61.9 (52.3, 70.9)  8, 7.1 (3.1, 13.5)  74, 67.3 (57.7, 75.9)  11, 10.1 (5.1, 17.3) | 6, 5.5  48, 42.5  6, 5.3  43, 39.1  10.1 | 1, 0.9  19, 16.8  2, 1.8  29, 19.1  0, 0 | 0, 0  3, 2.7  0, 0  9, 8.2  0, 0 | 0, 0  0, 0  0, 0  1, 0.9  0, 0 |
| Pain | 1 | A  B  C  D  E  B+C  D+E | 0/0  5/50  5/50  25/50  25/50  5/50  25/50 | 114  114  117  113  117  231  230 | 2, 1.8 (0.2, 6.2)  20, 17.5 (11.1, 25.8)  20, 17.1 (10.8, 25.2)  21, 18.6 (11.9, 27.0)  36, 30.8 (22.6, 40.0)  40, 17.3 (12.7,22.8)  57, 24.8 (19.3,30.9) | 2, 1.8  19, 16.7  20, 17.1  21, 18.6  36, 30.8  39, 16.9  57, 24.8 | 0, 0  1, 0.9  0, 0  0, 0  0, 0  1, 0.4  0, 0 | 0, 0  0, 0  0, 0  0, 0  0, 0  0, 0  0, 0 | 0, 0  0, 0  0, 0  0, 0  0, 0  0, 0  0, 0 |
|  | 2 | A  B  C  D  E | 0/0  5/50  0/0  25/50  0/0 | 110  113  113  110  109 | 3, 2.7 (0.6, 7.8)  46, 40.7 (31.6, 50.4)  5, 4.4 (1.5, 10.0)  43, 39.1 (29.9, 48.9)  6, 5.5 (2.0, 11.6) | 3, 2.7  45, 39.8  5, 4.4  34, 30.9  6, 5.5 | 0, 0  0, 0  0, 0  8, 7.3  0, 0 | 0, 0  1, 0.9  0, 0  0, 0  0, 0 | 0, 0  0, 0  0, 0  1, 0.9  0, 0 |
| Erythema | 1 | A  B  C  D  E  B+C  D+E | 0/0  5/50  5/50  25/50  25/50  5/50  25/50 | 114  114  117  113  117  231  230 | 0, 0 (0.0, 3.2)  0, 0 (0.0, 3.2)  1, 0.9 (0.0, 4.7)  1, 0.9 (0.0, 4.8)  1, 0.9 (0.0, 4.7)  1, 0.4 (0.0, 2.4)  2, 0.9 (0.1, 3.1) | 0, 0  0, 0  1, 0.9  0, 0  0, 0  1, 0.4  0, 0 | 0, 0  0, 0  0, 0  1, 0.9  1, 0.9  0, 0  2, 0.9 | 0, 0  0, 0  0, 0  0, 0  0, 0  0, 0  0, 0 | 0, 0  0, 0  0, 0  0, 0  0, 0  0, 0  0, 0 |
|  | 2 | A  B  C  D  E | 0/0  5/50  0/0  25/50  0/0 | 110  113  113  110  109 | 0, 0 (0.0, 3.3)  6, 5.3 (2.0, 11.2)  0, 0 (0.0, 3.2)  17, 15.5 (9.3, 23.6)  0, 0 (0.0, 3.3) | 0, 0  2, 1.8  0, 0  3, 2.7  0, 0 | 0, 0  3, 2.7  0, 0  8, 7.3  0, 0 | 0, 0  1, 0.9  0, 0  6, 5.5  0, 0 | 0, 0  0, 0  0, 0  0, 0  0, 0 |
| Swelling | 1 | A  B  C  D  E  B+C  D+E | 0/0  5/50  5/50  25/50  25/50  5/50  25/50 | 114  114  117  113  117  231  230 | 0, 0 (0.0, 3.2)  0, 0 (0.0, 3.2)  1, 0.9 (0.0, 4.7)  0, 0 (0.0, 3.2)  3, 2.6 (0.5, 7.3)  1, 0.4 (0.0, 2.4)  3, 1.3 (0.3, 3.8) | 0, 0  0, 0  0, 0  0, 0  1, 0.9  0, 0  1, 0.4 | 0, 0  0, 0  1, 0.9  0, 0  2, 1.7  1, 0.4  2, 0.9 | 0, 0  0, 0  0, 0  0, 0  0, 0  0, 0  0, 0 | 0, 0  0, 0  0, 0  0, 0  0, 0  0, 0  0, 0 |
|  | 2 | A  B  C  D  E | 0/0  5/50  0/0  25/50  0/0 | 110  113  113  110  109 | 0, 0 (0.0, 3.3)  9, 8.0 (3.7, 14.6)  0, 0 (0.0, 3.2)  11, 10.0 (5.1, 17.2)  0, 0 (0.0, 3.3) | 0, 0  4, 3.5  0, 0  3, 2.7  0, 0 | 0, 0  5, 4.4  0, 0  5, 4.5  0, 0 | 0, 0  0, 0  0, 0  3, 2.7  0, 0 | 0, 0  0, 0  0, 0  0, 0  0, 0 |
| Tenderness | 1 | A  B  C  D  E  B+C  D+E | 0/0  5/50  5/50  25/50  25/50  5/50  25/50 | 114  114  117  113  117  231  230 | 12, 10.5 (5.6, 17.7)  39, 34.2 (25.6, 43.7)  37, 31.6 (23.3, 40.9)  46, 40.7 (31.6, 50.4)  66, 56.4 (46.9, 65.6)  76, 32.9 (26.9, 39.4)  112, 48.7 (42.1, 55.4) | 11, 9.6  36, 31.6  32, 27.4  40, 35.4  49, 41.9  68, 29.4  89, 38.7 | 1, 0.9  3, 2.6  5, 4.3  6, 5.3  16, 13.7  8, 3.5  22, 9.6 | 0, 0  0, 0  0, 0  0, 0  1, 0.9  0, 0  1, 0.4 | 0, 0  0, 0  0, 0  0, 0  0, 0  0, 0  0, 0 |
|  | 2 | A  B  C  D  E | 0/0  5/50  0/0  25/50  0/0 | 110  113  113  110  109 | 6, 5.5 (2.0, 11.5)  62, 54.9 (45.2, 64.2)  5, 4.4 (1.5, 10.0)  68, 61.8 (52.1, 70.9)  9, 8.3 (3.8, 15.1) | 5, 4.5  43, 38.1  3, 2.7  46, 41.8  9, 8.3 | 1, 0.9  18, 15.9  2, 1.8  18, 16.4  0, 0 | 0, 0  1, 0.9  0, 0  3, 2.7  0, 0 | 0, 0  0, 0  0, 0  1, 0.9  0, 0 |
| Any solicited systemic AE | 1 | A  B  C  D  E  B+C  D+E | 0/0  5/50  5/50  25/50  25/50  5/50  25/50 | 113  116  117  113  117  233  230 | 36, 31.9 (23.4, 41.3)  44, 37.9 (29.1, 47.4)  36, 30.8 (22.6, 40.0)  33, 29.2 (21.0, 38.5)  40, 34.2 (25.7, 43.5)  80, 34.3 (28.3, 40.8)  73, 31.7 (25.8, 38.2) | 23, 20.4  26, 22.4  19, 16.2  24, 21.2  28, 23.1  45, 19.3  52, 22.6 | 12, 10.6  14, 12.1  15, 12.8  7, 6.2  12, 10.3  29, 12.4  19, 8.3 | 0, 0  4, 3.4  2, 1.7  2, 1.8  0, 0  6, 2.6  2, 0.9 | 1, 0.9  0, 0  0, 0  0, 0  0.9  0, 0  0, 0 |
|  | 2 | A  B  C  D  E | 0/0  5/50  0/0  25/50  0/0 | 109  113  113  110  108 | 24, 22.0 (14.6, 31.0)  44, 38.9 (29.9, 48.6)  25, 22.1 (14.9, 30.9)  56, 50.9 (41.2, 60.6)  22, 20.4 (13.2, 29.2) | 16, 14.7  22, 19.5  15, 13.3  23, 20.9  14, 13.0 | 7, 6.4  19, 16.8  8, 7.1  28, 25.5  8, 7.4 | 1, 0.9  3, 2.7  2, 1.8  4, 3.6  0, 0 | 0, 0  0, 0  0, 0  0.9  0, 0 |
| Joint pain/ Arthralgia | 1 | A  B  C  D  E  B+C  D+E | 0/0  5/50  5/50  25/50  25/50  5/50  25/50 | 113  116  117  113  117  233  230 | 3, 2.7 (0.6, 7.6)  6, 5.2 (1.9, 10.9)  13, 11.1 (6.1, 18.3)  4, 3.5 (1.0, 8.8)  10, 8.5 (4.2, 15.2)  19, 8.2 (5.0, 12.4)  14, 6.1 (3.4, 10.0) | 3, 2.7  1, 0.9  7, 6.0  4, 3.5  5, 4.3  8, 3.4  9, 3.9 | 0, 0  4, 3.4  6, 5.1  0, 0  5, 4.3  10, 4.3  5, 3.2 | 0, 0  1, 0.9  0, 0  0, 0  0, 0  1, 0.4  0, 0 | 0, 0  0, 0  0, 0  0, 0  0, 0  0, 0  0, 0 |
|  | 2 | A  B  C  D  E | 0/0  5/50  0/0  25/50  0/0 | 109  113  113  110  108 | 2, 1.8 (0.2, 6.5)  12, 10.6 (5.6, 17.8)  4, 3.5 (1.0, 8.8)  14, 12.7 (7.1, 20.4)  4, 3.7 (1.0, 9.2) | 0, 0  6, 5.3  1, 0.9  5, 4.5  2, 1.9 | 2, 1.8  4, 3.5  3, 2.7  9, 8.2  2, 1.9 | 0, 0  2, 1.8  0, 0  0, 0  0, 0 | 0, 0  0, 0  0, 0  0, 0  0, 0 |
| Fatigue | 1 | A  B  C  D  E  B+C  D+E | 0/0  5/50  5/50  25/50  25/50  5/50  25/50 | 113  116  117  113  117  233  230 | 20, 17.7 (11.2, 26.0)  22, 19.0 (12.3, 27.3)  21, 17.9 (11.5, 26.1)  12, 10.6 (5.6, 17.8)  16, 13.7 (8.0, 21.3)  43, 18.5 (13.7, 24.0)  28, 12.2 (8.2, 17.1) | 11, 9.7  9, 7.8  10, 8.5  5, 4.4  8, 6.8  19, 8.2  13, 5.7 | 9, 8.0  11, 9.5  9, 7.7  6, 5.3  8, 6.8  20, 8.6  14, 6.1 | 0, 0  2, 1.7  2, 1.7  1, 0.9  0, 0  4, 1.7  1, 0.4 | 0, 0  0, 0  0, 0  0, 0  0, 0  0, 0  0, 0 |
|  | 2 | A  B  C  D  E | 0/0  5/50  0/0  25/50  0/0 | 109  113  113  110  108 | 11, 10.1 (5.1, 17.3)  26, 23.0 (15.6, 31.9)  13, 11.5 (6.3, 18.9)  36, 32.7 (24.1, 42.3)  11, 10.2 (5.2, 17.5) | 6, 5.5  9, 8.0  7, 6.2  11, 10.0  4, 3.7 | 4, 3.7  17, 15.0  5, 4.4  22, 20.0  7, 6.5 | 1, 0.9  0, 0  1, 0.9  3, 2.7  0, 0 | 0, 0  0, 0  0, 0  0, 0  0, 0 |
| Malaise | 1 | A  B  C  D  E  B+C  D+E | 0/0  5/50  5/50  25/50  25/50  5/50  25/50 | 113  116  117  113  117  233  230 | 9, 8.0 (3.7, 14.6)  18, 15.5 (9.5, 23.4)  13, 11.1 (6.1, 18.3)  5, 4.4 (1.5, 10.0)  12, 10.3 (5.4, 17.2)  31, 13.3 (9.2, 18.4)  17, 5.0 (4.4, 11.6) | 3, 2.7  10, 8.6  7, 6.0  3, 2.7  9, 7.7  17, 7.3  11, 4.8 | 5, 4.4  6, 5.2  4, 3.4  1, 0.9  3, 2.6  10, 4.3  4, 1.7 | 0, 0  2, 1.7  2, 1.7  1, 0.9  0, 0  4, 1.7  1, 0.4 | 1, 0.9  0, 0  0, 0  0, 0  0, 0  0, 0  0, 0 |
|  | 2 | A  B  C  D  E | 0/0  5/50  0/0  25/50  0/0 | 109  113  113  110  108 | 8, 7.3 (3.2, 14.0)  23, 20.4 (13.4, 29.0)  7, 6.2 (2.5, 12.3)  18, 16.4 (10.0, 24.6)  4, 3.7 (1.0, 9.2) | 5, 4.6  8, 7.1  3, 2.7  3, 2.7  3, 2.8 | 3, 2.8  14, 12.4  3, 2.7  13, 11.8  1, 0.9 | 0, 0  1, 0.9  1, 0.9  2, 1.8  0, 0 | 0, 0  0, 0  0, 0  0, 0  0, 0 |
| Temperature/ Fever | 1 | A  B  C  D  E  B+C  D+E | 0/0  5/50  5/50  25/50  25/50  5/50  25/50 | 112  116  117  113  117  233  230 | 3, 2.7 (0.6, 7.6)  4, 3.4 (0.9, 8.6)  3, 2.6 (0.5, 7.3)  2, 1.8 (0.2, 6.2)  1, 0.9 (0.0, 4.7)  7, 3.0 (1.2, 6.1)  3, 1.3 (0.3, 3.8) | 1, 0.9  2, 1.7  2, 1.7  0, 0  0, 0  4, 1.7  0, 0 | 2, 1.8  0, 0  1, 0.9  1, 0.9  1, 0.9  1, 0.4  2, 0.9 | 0, 0  2, 1.7  0, 0  1, 0.9  0, 0  2, 0.9  1, 0.4 | 0, 0  0, 0  0, 0  0, 0  0, 0  0, 0  0, 0 |
|  | 2 | A  B  C  D  E | 0/0  5/50  0/0  25/50  0/0 | 109  112  113  108  107 | 1, 0.9 (0.0, 5.0)  2, 1.8 (0.2, 6.3)  0, 0 (0.0, 3.2)  4, 3.7 (1.0, 9.2)  1, 0.9 (0.0, 5.1) | 1, 0.9  2, 1.8  0, 0  3, 2.8  0, 0 | 0, 0  0, 0  0, 0  1, 0.9  1, 0.9 | 0, 0  0, 0  0, 0  0, 0  0, 0 | 0, 0  0, 0  0, 0  0, 0  0, 0 |
| Headache | 1 | A  B  C  D  E  B+C  D+E | 0/0  5/50  5/50  25/50  25/50  5/50  25/50 | 113  116  117  113  117  233  230 | 17, 15.0 (9.0, 23.0)  19, 16.4 (10.2, 24.4)  12, 10.3 (5.4, 17.2)  13, 11.5 (6.3, 18.9)  12, 10.3 (5.4, 17.2)  31, 13.3 (9.2, 18.4)  25, 10.9 (7.2, 15.6) | 15, 13.3  15, 12.9  9, 7.7  12, 10.6  11, 9.4  24, 10.3  23, 10.0 | 2, 1.8  4, 3.4  3, 2.6  1, 0.9  1, 0.9  7, 3.0  2, 0.9 | 0, 0  0, 0  0, 0  0, 0  0, 0  0, 0  0, 0 | 0, 0  0, 0  0, 0  0, 0  0, 0  0, 0  0, 0 |
|  | 2 | A  B  C  D  E | 0/0  5/50  0/0  25/50  0/0 | 109  113  113  110  108 | 12, 11.0 (5.8, 18.4)  17, 15.0 (9.0, 23.0)  12, 10.6 (5.6, 17.8)  28, 25.5 (17.6, 34.6)  12, 11.1 (5.9, 18.6) | 11, 10.1  10, 8.8  9, 8.0  14, 12.7  11, 10.2 | 1, 0.9  6, 5.3  2, 1.8  13, 11.8  1, 0.9 | 0, 0  1, 0.9  1, 0.9  1, 0.9  0, 0 | 0, 0  0, 0  0, 0  0, 0  0, 0 |
| Muscle pain/ Myalgia | 1 | A  B  C  D  E  B+C  D+E | 0/0  5/50  5/50  25/50  25/50  5/50  25/50 | 113  116  117  113  117  233  230 | 9, 8.0 (3.7, 14.6)  16, 13.8 (8.1, 21.4)  19, 16.2 (10.1, 24.2)  12, 10.6 (5.6, 17.8)  21, 17.9 (11.5, 26.1)  35, 15.0 (10.7, 20.3)  33, 14.3 (10.1, 19.6) | 6, 5.3  12, 10.3  16, 13.7  11, 9.7  18, 15.4  28, 12.0  29, 12.6 | 3, 2.7  3, 2.6  3, 2.6  1, 0.9  3, 2.6  6, 2.6  4, 1.7 | 0, 0  1, 0.9  0, 0  0, 0  0, 0  1, 0.4  0, 0 | 0, 0  0, 0  0, 0  0, 0  0, 0  0, 0  0, 0 |
|  | 2 | A  B  C  D  E | 0/0  5/50  0/0  25/50  0/0 | 109  113  113  110  108 | 1, 0.9 (0.0, 5.0)  22, 19.5 (12.6, 28.0)  4, 3.5 (1.0, 8.8)  32, 29.1 (20.8, 38.5)  3, 2.8 (0.6, 7.9) | 0, 0  10, 8.8  2, 1.8  15, 13.6  1, 0.9 | 1, 0.9  11, 9.7  2, 1.8  16, 14.5  2, 1.9 | 0, 0  1, 0.9  0, 0  0, 0  0, 0 | 0, 0  0, 0  0, 0  1, 0.9  0, 0 |
| Nausea or vomiting | 1 | A  B  C  D  E  B+C  D+E | 0/0  5/50  5/50  25/50  25/50  5/50  25/50 | 113  116  117  113  117  233  230 | 2, 1.8 (0.2, 6.2)  6, 5.2 (1.9, 10.9)  4, 3.4 (0.9, 8.5)  3, 2.7 (0.6, 7.6)  3, 2.6 (0.5, 7.3)  10, 4.3 (2.1, 7.8)  6, 2.6 (1.0, 5.6) | 2, 1.8  3, 2.6  3, 2.6  3, 2.7  3, 2.6  6, 2.6  6, 2.6 | 0, 0  3, 2.6  1, 0.9  0, 0  0, 0  3, 1.7  0, 0 | 0, 0  0, 0  0, 0  0, 0  0, 0  0, 0  0, 0 | 0, 0  0, 0  0, 0  0, 0  0, 0  0, 0  0, 0 |
|  | 2 | A  B  C  D  E | 0/0  5/50  0/0  25/50  0/0 | 109  113  113  110  108 | 1, 0.9 (0.0, 5.0)  7, 6.2 (2.5, 12.3)  1, 0.9 (0.0, 4.8)  8, 7.3 (3.2, 13.8)  1, 0.9 (0.0, 5.1) | 1, 0.9  6, 5.3  0, 0  6, 5.5  1, 0.9 | 0, 0  1, 0.9  1, 0.9  2, 1.8  0, 0 | 0, 0  0, 0  0, 0  0, 0  0, 0 | 0, 0  0, 0  0, 0  0, 0  0, 0 |

Abbreviations: AE = adverse event; CI = confidence interval; SARS‑CoV-2 rS = severe acute respiratory syndrome coronavirus 2 recombinant spike protein nanoparticle vaccine.

* N denotes number of participants assessed.

† Group A (placebo): 0 µg rSARS-CoV-2/0 µg Matrix-M1 adjuvant on first vaccination; 0 µg rSARS-CoV-2/0 µg Matrix-M1 adjuvant on second vaccination. Group B: 5 µg rSARS-CoV-2/50 µg Matrix-M1 adjuvant on first vaccination; 5 µg rSARS-CoV-2/50 µg Matrix-M1 adjuvant on second vaccination. Group C: 5 µg rSARS-CoV-2/50 µg Matrix-M1 adjuvant on first vaccination; 0 µg rSARS-CoV-2/0 µg Matrix-M1 adjuvant on second vaccination. Group D: 25 µg rSARS-CoV-2/50 µg Matrix-M1 adjuvant on first vaccination; 25 µg rSARS-CoV-2/50 µg Matrix-M1 adjuvant on second vaccination. Group E: 25 µg rSARS-CoV-2/50 µg Matrix-M1 adjuvant on first vaccination; 0 µg rSARS-CoV-2/0 µg Matrix-M1 adjuvant on second vaccination.

### Table H. Unsolicited Adverse Events by System Organ Class and Preferred Term Reported in 1% or More Participants in Any Group Through 35 Days After First Vaccination (Safety Analysis Set).*

| **Group**  AGEU | **A** | **B** | **C** | **D** | **E** | **Total**  **1283** |
| --- | --- | --- | --- | --- | --- | --- |
| **Study treatments Dose 1 / Dose 2** | **Placebo / Placebo** | **5 µg + M1 / 5 µg + M1** | **5 µg + M1 / Placebo** | **25 µg + M1 / 25 µg + M1** | **25 µg + M1 / Placebo** |  |
| **System Organ Class/Preferred Term† N** | **255** | **258** | **256** | **259** | **255** |  |
| Any AE‡ | 42 (16.5) | 51 (19.8) | 35 (13.7) | 52 (20.1) | 43 (16.9) | 223 (17.4) |
| Infections and infestations | 9 (3.5) | 8 (3.1) | 7 (2.7) | 10 (3.9) | 6 (2.4) | 40 (3.1) |
| Urinary tract infection | 2 (0.8) | 2 (0.8) | 1 (0.4) | 2 (0.8) | 3 (1.2) | 10 (0.8) |
| Injury, poisoning, and procedural complications | 7 (2.7) | 7 (2.7) | 7 (2.7) | 5 (1.9) | 6 (2.4) | 32 (2.5) |
| Gastrointestinal disorders | 7 (2.7) | 9 (3.5) | 3 (1.2) | 6 (2.3) | 6 (2.4) | 31 (2.4) |
| General disorders and administration site conditions | 6 (2.4) | 7 (2.7) | 1 (0.4) | 13 (5.0) | 2 (0.8) | 29 (2.3) |
| Injection site pruritus | 0 | 3 (1.2) | 0 | 5 (1.9) | 0 | 8 (0.6) |
| Musculoskeletal and connective tissue disorders | 5 (2.0) | 8 (3.1) | 5 (2.0) | 5 (1.9) | 4 (1.6) | 27 (2.1) |
| Arthralgia | 2 (0.8) | 3 (1.2) | 0 | 1 (0.4) | 1 (0.4) | 7 (0.5) |
| Nervous system disorders | 4 (1.6) | 5 (1.9) | 4 (1.6) | 6 (2.3) | 2 (0.8) | 21 (1.6) |
| Headache | 2 (0.8) | 3 (1.2) | 1 (0.4) | 2 (0.8) | 1 (0.4) | 9 (0.7) |
| Respiratory, thoracic, and mediastinal disorders | 3 (1.2) | 4 (1.6) | 3 (1.2) | 5 (1.9) | 4 (1.6) | 19 (1.5) |
| Skin and subcutaneous tissue disorders | 2 (0.8) | 3 (1.2) | 2 (0.8) | 3 (1.2) | 8 (3.1) | 18 (1.4) |
| Blood and lymphatic system disorders | 1 (0.4) | 3 (1.2) | 1 (0.4) | 2 (0.8) | 2 (0.8) | 9 (0.7) |
| Lymphadenopathy | 1 (0.4) | 3 (1.2) | 1 (0.4) | 1 (0.4) | 2 (0.8) | 8 (0.6) |
| Vascular disorders | 2 (0.8) | 2 (0.8) | 1 (0.4) | 0 | 3 (1.2) | 8 (0.6) |
| Hypertension | 1 (0.4) | 2 (0.8) | 0 | 0 | 3 (1.2) | 6 (0.5) |

Abbreviations: AE = adverse event; MedDRA = *Medical Dictionary for Regulatory Activities;* M1 = Matrix M1 adjuvant 50 µg.

* N denotes number of participants assessed.

† Adverse events were coded using MedDRA, version 23.0.

‡ Based on all AEs.

**[Supporting information (Supplementary Appendix) captions and footnotes]**

**Table A. Toxicity Grading Scales for Solicited Local and Systemic Adverse Events – Modified From FDA Toxicity Grading Scale for Clinical Abnormalities.**

Abbreviations: ER = emergency room; IV = intravenous.

* The measurements should be recorded as a continuous variable.

† These events are not subject reported through the electronic diary and will be monitored through the adverse event pages of the study database.

‡ Oral temperature if subject collected, sites may collect temperature using local clinic practices/devices. Toxicity grade will be derived.

**Table B. Adverse Events of Special Interest Relevant to COVID-19.***

* COVID-19 disease manifestations associated with more severe presentation and decompensation with consideration of enhanced disease potential. The current listing is based on Safety Platform for Emergency Vaccines (SPEAC) D2.3 Priority List of Adverse Events of Special Interest: COVID-19 (SPEAC 2020).

† Cytokine release syndrome related to COVID-19 disease is a disorder characterized by nausea, headache, tachycardia, hypotension, rash, and/or shortness of breath.

**Table C. Potential Immune-Mediated Medical Conditions.**

Abbreviation: MEDRA = *Medical Dictionary for Regulatory Activities,* version 23.0.

* For Hashimoto’s thyroiditis: new onset only.

**Table D. Overall Summary of Solicited and Unsolicited Adverse Events Following Vaccination of SARS-CoV-2 rS With Matrix-M1 Adjuvant in Adult Participants 18 to 84 Years of Age (Safety Analysis Set).**

Abbreviations: AE = adverse event; AESI = adverse event of special interest; COVID-19 = coronavirus disease 2019; FDA = US Food and Drug Administration; MAAE = medically attended adverse event; PIMMC = potential immune-mediated medical conditions; SARS‑CoV-2 rS = severe acute respiratory syndrome coronavirus 2 recombinant spike protein nanoparticle vaccine.

* Includes solicited AEs reported by participants (via diary or spontaneously) with a recorded start date within 7 days post each vaccination window.

Note: Data are presented as number and percentage (n [%]) of participants. CIs not calculated.

**Table E. Percentage of All Participants (18 to 84 years) Experiencing Solicited Local and Systemic Adverse Events by Symptom, Vaccination Dose, Vaccine Group, and Maximum Toxicity Grade (Safety Analysis Set).***

Abbreviations: AE = adverse event; CI = confidence interval; SARS‑CoV-2 rS = severe acute respiratory syndrome coronavirus 2 recombinant spike protein nanoparticle vaccine.

* N denotes number of participants assessed.

† Group A (placebo): 0 µg rSARS-CoV-2/0 µg Matrix-M1 adjuvant on first vaccination; 0 µg rSARS-CoV-2/0 µg Matrix-M1 adjuvant on second vaccination. Group B: 5 µg rSARS-CoV-2/50 µg Matrix-M1 adjuvant on first vaccination; 5 µg rSARS-CoV-2/50 µg Matrix-M1 adjuvant on second vaccination. Group C: 5 µg rSARS-CoV-2/50 µg Matrix-M1 adjuvant on first vaccination; 0 µg rSARS-CoV-2/0 µg Matrix-M1 adjuvant on second vaccination. Group D: 25 µg rSARS-CoV-2/50 µg Matrix-M1 adjuvant on first vaccination; 25 µg rSARS-CoV-2/50 µg Matrix-M1 adjuvant on second vaccination. Group E: 25 µg rSARS-CoV-2/50 µg Matrix-M1 adjuvant on first vaccination; 0 µg rSARS-CoV-2/0 µg Matrix-M1 adjuvant on second vaccination.

**Table F. Percentage of Younger Adults (18 to 59 Years) Experiencing Solicited Local and Systemic Adverse Events by Symptom, Vaccination Dose, Vaccine Group, and Maximum Toxicity Grade (Safety Analysis Set).***

Abbreviations: AE = adverse event; CI = confidence interval; SARS‑CoV-2 rS = severe acute respiratory syndrome coronavirus 2 recombinant spike protein nanoparticle vaccine.

* N denotes number of participants assessed.

† Group A (placebo): 0 µg rSARS-CoV-2/0 µg Matrix-M1 adjuvant on first vaccination; 0 µg rSARS-CoV-2/0 µg Matrix-M1 adjuvant on second vaccination. Group B: 5 µg rSARS-CoV-2/50 µg Matrix-M1 adjuvant on first vaccination; 5 µg rSARS-CoV-2/50 µg Matrix-M1 adjuvant on second vaccination. Group C: 5 µg rSARS-CoV-2/50 µg Matrix-M1 adjuvant on first vaccination; 0 µg rSARS-CoV-2/0 µg Matrix-M1 adjuvant on second vaccination. Group D: 25 µg rSARS-CoV-2/50 µg Matrix-M1 adjuvant on first vaccination; 25 µg rSARS-CoV-2/50 µg Matrix-M1 adjuvant on second vaccination. Group E: 25 µg rSARS-CoV-2/50 µg Matrix-M1 adjuvant on first vaccination; 0 µg rSARS-CoV-2/0 µg Matrix-M1 adjuvant on second vaccination.

**Table G. Percentage of Older Adults (60 to 84 Years) Experiencing Solicited Local and Systemic Adverse Events by Symptom, Vaccination Dose, Vaccine Group, and Maximum Toxicity Grade (Safety Analysis Set).***

Abbreviations: AE = adverse event; CI = confidence interval; SARS‑CoV-2 rS = severe acute respiratory syndrome coronavirus 2 recombinant spike protein nanoparticle vaccine.

* N denotes number of participants assessed.

† Group A (placebo): 0 µg rSARS-CoV-2/0 µg Matrix-M1 adjuvant on first vaccination; 0 µg rSARS-CoV-2/0 µg Matrix-M1 adjuvant on second vaccination. Group B: 5 µg rSARS-CoV-2/50 µg Matrix-M1 adjuvant on first vaccination; 5 µg rSARS-CoV-2/50 µg Matrix-M1 adjuvant on second vaccination. Group C: 5 µg rSARS-CoV-2/50 µg Matrix-M1 adjuvant on first vaccination; 0 µg rSARS-CoV-2/0 µg Matrix-M1 adjuvant on second vaccination. Group D: 25 µg rSARS-CoV-2/50 µg Matrix-M1 adjuvant on first vaccination; 25 µg rSARS-CoV-2/50 µg Matrix-M1 adjuvant on second vaccination. Group E: 25 µg rSARS-CoV-2/50 µg Matrix-M1 adjuvant on first vaccination; 0 µg rSARS-CoV-2/0 µg Matrix-M1 adjuvant on second vaccination.

**Table H. Unsolicited Adverse Events by System Organ Class and Preferred Term Reported in 1% or More Participants in Any Group Through 35 Days After First Vaccination (Safety Analysis Set).***

Abbreviations: AE = adverse event; MedDRA = *Medical Dictionary for Regulatory Activities;* M1 = Matrix M1 adjuvant 50 µg.

* N denotes number of participants assessed.

† Adverse events were coded using MedDRA, version 23.0.

‡ Based on all AEs.

[Fig A is in Word – Fig A appears after Table C]

**Fig A. Vaccine Regimens and Key Trial Assessments.** Shown in are the planned randomization schema and associated vaccine regimens administered in the trial (Panel A), along with timing of the key safety and immunogenicity assessments (Panel B).

Abbreviations: CMI = cell-mediated immunity; SARS‑CoV-2 rS = severe acute respiratory syndrome coronavirus 2 recombinant spike protein nanoparticle vaccine.
