# Supplementary material for: Different dose regimens of a SARS-CoV-2 recombinant spike protein vaccine (NVX-CoV2373) in younger and older adults: A phase 2 randomized placebo-controlled trial
Source: PLoS Med. 2021 Oct 1;18(10):e1003769. doi: 10.1371/journal.pmed.1003769 (PMC8486115; doi:10.1371/journal.pmed.1003769)
Supplement: Attachment — Submitted filename: Review_21-01479R1.docx [file pmed.1003769.s003.docx]

**Review of ‘Evaluation of a SARS-CoV-2 vaccine NVX-CoV2373 in younger and older adults’**

The manuscript describes the safety and immunogenicity results of a ph1-2 trial of a SARS-CoV-2 vaccine in younger and older adults in Australia and the US, a topic with wide appeal to the general readership of PLOS Medicine.

The manuscript is well-written but the results and findings need to be better quantified and supported by statistical analyses.

The abstract contains various statements about greater reactogenicity after the second vs first vaccine and in younger vs older adults but no estimates/CIs/p-values are provided as supportive evidence. The abstract should also quantify what is meant by “predominantly mild to moderate” reactogenicity by including the observed rates.

The Methods section states that the protocol and statistical analysis plan are available but these were not submitted with the manuscript and couldn’t be reviewed.

It is unclear how the sample size of “up to 750 participants” was selected. What determined the final sample size and what was the minimum sample size? Sample size calculations should be provided. It is not sufficient to state that there is more than adequate power to detect differences in IgG responses and safety outcomes between vaccine groups- this depends very much on the differences of interest, the variability of the assay and the expected levels of response. In addition, it seems that the goal was to establish non-inferiority of the lower dose regimen- the manuscript claims that the two-dose regimen of 5μg is as tolerable and as immunogenic as the two-dose regimen of 25μg. Statements such as these require statistical analyses that support ruling out a non-inferiority margin of >= x so that one can claim the difference in the two regimens is at most x. One can’t claim that there is no difference between regimens after failing to find a significant difference. If the sample size is small, one may not see a difference but this is not because a difference doesn’t exist but rather that the trial was underpowered to detect it. Without power calculations, the reader can’t make this determination.

It would be of interest to readers to see a finer categorization of age in Table 1 to get a sense of how many older (>75) participants were enrolled.

The results on safety outcomes should include 95%CIs and p-values when stating any group differences.

Please list reasons for exclusion of 28 individuals in the second box of the CONSORT diagram.

Please specify whether Spearman or Pearson correlations are reported in Fig 4 and whether the reported 95%CIs are 1-sided or 2-sided.
