# Supplementary material for: Different dose regimens of a SARS-CoV-2 recombinant spike protein vaccine (NVX-CoV2373) in younger and older adults: A phase 2 randomized placebo-controlled trial
Source: PLoS Med. 2021 Oct 1;18(10):e1003769. doi: 10.1371/journal.pmed.1003769 (PMC8486115; doi:10.1371/journal.pmed.1003769)
Supplement: Attachment — Submitted filename: D-21-01479R1_PLOS Medicine_Response_Ltr_14June2021.docx [file pmed.1003769.s004.docx]

Beryne Odeny, MD, MPH
*PLOS Medicine*

15 June 2021

Dear Dr. Odeny:

Thank you for your review of our manuscript entitled “A phase 2 randomized placebo-controlled trial of different dose regimens of a SARS-CoV-2 recombinant spike protein vaccine (NVX-CoV2373) in younger and older adults” (PMEDICINE-D-21-01479R1). We appreciate the comments from your editors and the independent reviewers, and we have provided point-by-point responses to the comments in the table below.

According to your instructions, we have submitted two versions of the manuscript revision – a clean version and a marked-up version, which shows the changes we have made. We have also provided a revised version of the supplementary appendix, and we have included the CONSORT checklist.

Thank you for considering our manuscript for publication in *PLOS Medicine.*

Sincerely,

Neil Formica, MBBS

On behalf of the authors

**First round of review comments and responses for PMEDICINE-D-21-01479R1**

| **FEEDBACK FROM:** | **RESPONSES** |
| --- | --- |
| **PLOS MEDICINE Editorial group** |  |
| To enhance the reproducibility of your results, we recommend that you deposit your **laboratory protocols** in protocols.io, where a protocol can be assigned its own identifier (DOI) such that it can be cited independently in the future.  Additionally, PLOS ONE offers an option to publish peer-reviewed **clinical study protocols**. Read more information on sharing protocols at https://plos.org/protocols?utm_medium=editorial-email&utm_source=authorletters&utm_campaign=protocols | Novavax will provide a redacted protocol and statistical analysis plan related to the analysis for this publication to be included in supplementary materials upon acceptance of the manuscript.  Laboratory protocols will not be submitted at this time since assay development and refinement is ongoing. |
| Please ensure that the paper adheres to the **PLOS Data Availability Policy** (see http://journals.plos.org/plosmedicine/s/data-availability), which requires that all data underlying the study's findings be provided in a repository or as Supporting Information. For data residing with a third party, authors are required to provide instructions with contact information for obtaining the data. PLOS journals do not allow statements supported by "data not shown" or "unpublished results." For such statements, authors must provide supporting data or cite public sources that include it. | The supplementary Appendix includes additional data supporting the study’s findings. |
| **Comments from Reviewer 1 (Editorial Reviewer)** |  |
| 1) Please revise your title to indicate that this is an implementation science study. Your title must be nondeclarative and not a question. It should begin with main concept if possible. For example, please place the study design ("A phase II randomized placebo-controlled trial,") in the subtitle (i.e., after a colon). | The title of the manuscript has been revised to:  “A phase 2 randomized placebo-controlled trial of different dose regimens of a SARS-CoV-2 recombinant spike protein vaccine (NVX-CoV2373) in younger and older adults”. Page 1 lines 3-4. |
| 2) Abstract summary - At this stage, we ask that you reformat your non-technical Author Summary. The Author Summary should immediately follow the Abstract in your revised manuscript. This text is subject to editorial change and should be distinct from the scientific abstract. The summary should be accessible to a wide audience that includes both scientists and non-scientists. Please see our author guidelines for more information: https://journals.plos.org/plosmedicine/s/revising-your-manuscript#loc-author-summary. | We have provided an Author Summary, which follows the abstract. The Author Summary is written in a manner that allows it to be accessible to both scientists and non-scientists. Pages 3-4 lines 51-66. |
| 3) Abstract:  a) Please structure your abstract using the PLOS Medicine headings (Background, Methods and Findings, Conclusions). | The abstract has been re-formatted to follow the specific structure required by *PLOS Medicine.* Pages 2-3. |
| b) Please combine the Methods and Findings sections into one section, “Methods and findings”. Please ensure that all numbers presented in the abstract are present and identical to numbers presented in the main manuscript text. | We have combined the Methods and Findings sections into one section. We have checked that all numbers that appear in the abstract also appear in the main manuscript. Page 2. |
| c) Please report your abstract according to CONSORT for abstracts: http://www.consort-statement.org/extensions?ContentWidgetId=562 | The abstract provides the information required by CONSORT. Pages 2-3. |
| d) Please quantify the main results (with p values in addition to 95% CI). | CIs and p-values have been added to revised solicited AE tables in appendix, and incorporated in manuscript text. Such as Tables 2-3 in manuscript – pages – 14-17 and Tables S5a-5c in supplementary materials.  We chose CIs instead of p-values because p-values only indicate whether a result is statistically significant whereas CIs also tell the strength and direction of the significance |
| e) Please include a summary of adverse events if these were assessed in the study. | A formal summary of adverse events in the abstract is not conducive to concise expression. Summary of adverse events are described in the manuscript text in safety results and shown in Figure 2 and Tables S4 and S5. Table S4 (page 11 of supplement) is an overall summary of solicited and unsolicited adverse events added for this resubmission. |
| f) In the last sentence of the Abstract Methods and Findings section, please describe the main limitation(s) of the study's methodology. | Limitations have been provided as last sentence in the Abstract Methods and Findings section. In Abstract, p3, lines 42-45; in text: page 29, lines 482-486. |
| 4) Please complete the CONSORT checklist (CONSORT extension for early phase dose-finding clinical trials: https://www.equator-network.org/library/reporting-guidelines-under-development/reporting-guidelines-under-development-for-clinical-trials/ ). When completing the checklist, please use section and paragraph numbers, rather than page numbers. | We have completed the CONSORT checklist as requested. |
| 5) Please provide the following as Supplementary Information files: trial design, conduct, oversight, and analyses are provided in the protocol and statistical analysis plan | This information has been provided in the protocol (Version 8.0, 17 Dec 2020) and statistical analysis plan (V2.0, 02 Dec 2020).  The protocol and SAP have previously been provided to the journal, and versions with redaction of details of individuals or other confidential material will be provided for inclusion in the supplementary materials. |
| 6) How was race/ethnicity defined and by whom? Why was race/ethnicity considered important in this study and what it is believed to represent [eg, are differences being attributed to race/ethnicity? | Collection of Race and Ethnicity Data in Clinical Trials  Guidance for Industry and Food and Drug Administration Staff  Document issued on October 26, 2016  <https://www.fda.gov/media/75453/download>  Race/ethnicity are always reported in vaccine trials to provide information about the potential generalizability of results – and to allow regulators to assess whether the study population is sufficiently representative of their national populations.  However, routine race/ethnicity subgroup analyses are not routinely done but may be requested by regulators. |
| 7) In the Methods and Results section:  a) Please provide 95% CIs and p values for estimates in the main text and tables | The 95% CIs have been provided, but p-values are not included. We chose CIs instead of p-values because p-values only indicate whether a result is statistically significant whereas CIs also tell the strength and direction of the significance.  See Tables 2-3 in manuscript – pages 14-17 and Tables S5a-5c in supplementary materials. |
| b) When a p value is given, please specify the statistical test used to determine it. | p values are not included as previously described. |
| 8) Figures and tables:  a) Please indicate in the figure caption the meaning of the whiskers in Figures | The description of “whiskers” is in the figure legend:  “Whisker endpoints are equal to the maximum and minimum values below or above the median ± 1.5 times the IQR.” Page 26 – lines 414-415. |
| b) Please provide definitions for the following abbreviations: IC, AEs, EU/mL | Definitions have been provided. Page 19, line 265; Page 26, line 416; Page 45, line 691. |
| 9) Please replace "subject" with participant, patient, individual, or person. | The word “subject” has been replaced with appropriate word (participant or person). |
| 10) Under the “Discussion” section, 1st paragraph (line 337, 342), the term "trend" is used to refer to a nonsignificant P value. The term trend should be used only when the test for trend has been conducted. Please revise accordingly. | References to “trend” or terminology implying formal comparisons have been removed. |
| **Comments from Reviewer R1 [sent in separate Word document]** |  |
| The manuscript describes the safety and immunogenicity results of a ph1-2 trial of a SARS-CoV-2 vaccine in younger and older adults in Australia and the US, a topic with wide appeal to the general readership of PLOS Medicine.  The manuscript is well-written but the results and findings need to be better quantified and supported by statistical analyses. | We have revised sections of the manuscript so it is better quantified and supported by statistical analyses (see following responses). |
| The abstract contains various statements about greater reactogenicity after the second vs first vaccine and in younger vs older adults but no estimates/CIs/p-values are provided as supportive evidence. The abstract should also quantify what is meant by “predominantly mild to moderate” reactogenicity by including the observed rates. | We have reviewed the abstract and finalized it with some editorial changes. Pages 2-3. |
| The Methods section states that the protocol and statistical analysis plan are available but these were not submitted with the manuscript and couldn’t be reviewed. | The protocol and SAP have previously been provided to the journal, and versions with redaction of details of individuals or other confidential material will be provided for inclusion in the supplementary materials. |
| It is unclear how the sample size of “up to 750 participants” was selected. What determined the final sample size and what was the minimum sample size? Sample size calculations should be provided. It is not sufficient to state that there is more than adequate power to detect differences in IgG responses and safety outcomes between vaccine groups- this depends very much on the differences of interest, the variability of the assay and the expected levels of response.  In addition, it seems that the goal was to establish non-inferiority of the lower dose regimen- the manuscript claims that the two-dose regimen of 5μg is as tolerable and as immunogenic as the two-dose regimen of 25μg. Statements such as these require statistical analyses that support ruling out a non-inferiority margin of >= x so that one can claim the difference in the two regimens is at most x. One can’t claim that there is no difference between regimens after failing to find a significant difference. If the sample size is small, one may not see a difference but this is not because a difference doesn’t exist but rather that the trial was underpowered to detect it. Without power calculations, the reader can’t make this determination. | We have updated the Statistical Analysis section and added clarifying text to the manuscript. Page 10 – lines 177-192.  As per the protocol and SAP, the goal was not to establish non-inferiority – this was a descriptive dose-ranging and dose regimen phase 2 study.  Additional information on sample size calculations have been added. |
| It would be of interest to readers to see a finer categorization of age in Table 1 to get a sense of how many older (>75) participants were enrolled. | Table 1 now has a category of participants 75-84 years of age. Page 12. |
| The results on safety outcomes should include 95%CIs and p-values when stating any group differences. | CIs and p-values have been added to revised solicited AE tables in appendix, and incorporated in manuscript text. Tables S5a-5c in supplementary materials.  In several analyses, we chose CIs instead of p-values because p-values only indicate whether a result is statistically significant whereas CIs also tell the strength and direction of the significance. |
| Please list reasons for exclusion of 28 individuals in the second box of the CONSORT diagram. | The revised CONSORT diagram provides more details on inclusion and exclusion. Figure 1 – page 38, line 636. |
| Please specify whether Spearman or Pearson correlations are reported in Fig 4 and whether the reported 95%CIs are 1-sided or 2-sided. | The correlation plots were generated using a Pearson 2-sided approach. Figure 4, page 45, line 689. |
| **Comments from Reviewer 2 (Expert Independent Reviewer)** |  |
| This is a Phase 2 trial reporting the immunogenicity and reactogenicity profile of a matrix-M1 adjuvanted spike protein SARS-Cov-2 vaccine. This is not clear from the title of the paper which just mentions "evaluation" of a "SARS-CoV-2 vaccine NVX-CoV2373". Most readers will not be familiar with this name for the vaccine. It would help if the title indicated this was a Phase 2 trial and also the vaccine format, eg as per the Phase I NEJM preliminary report. | The title of the manuscript has been revised to:  “A phase 2 randomized placebo-controlled trial of different dose regimens of a SARS-CoV-2 recombinant spike protein vaccine (NVX-CoV2373) in younger and older adults” Page 1 – lines 3-4. |
| Can the authors clarify whether any of the 18-59 year olds in the current Phase 2 were also reported in the Phase 1 as the NEJM paper and this one both refer to the Phase1-2 trial with the Phase 2 component enrolling older adults. | We clarified the issue in the text – Phase 1 participants were not included in the Phase 2. Page 6 – lines 97-98. |
| Abstract: The abstract gives no idea of the numbers of subjects or CIs around the key immunogenicity results.  For example on page 2 line 41 it says that seroconversion rates in the wild type neutralisation assay are 100% in both age groups. However this is based on just 49 subjects across both age groups with no age breakdown given (Table S9) so the CIs around this estimate by age group will be quite wide. | Confidence intervals around assay results have been added to the abstract, and tables with confidence intervals for immunogenicity results have been added to the body of the manuscript. See main examples for Tables 2-3 in manuscript – pages – 14-17 and Tables S5a-5c in supplementary materials. |
| Also it is unclear how the subset tested for neutralising antibodies was selected. | Details on selection processes for the non-randomized neutralizing antibodies subset have been added. Page 9 – lines 163-166. |
| Introduction: Page 4 line 74 - what does "strong/high" levels mean in relation to a Th1 response? I could not find mention of "strong/high" in the NEJM paper to which this statement refers. | This statement was rewritten to remove reference to “strong/high” levels. |
| Fig 1 Consort diagram. It wasn't immediately clear to me what the 5/50 and 0/50 notation referred to. Can this be clarified in the text when there is the initial mention of the 5 study groups. | The CONSORT diagram has been revised and provides more clarity on the participants and the trial. Summary terminology for the treatment group antigen and adjuvant concentrations have been removed (eg. 5/50). Figure 1 – page 38, line 639 |
| Also from the supplementary appendix it appears that some of the groups will receive a third dose at 189 days which might merit a mention in the main text, eg discussion?. | Statement added in discussion that 6-month immune persistence and subgroups with 6-month booster are underway. Page 27 – lines 439-440. |
| Page 7 line 119. Does "within 7 days" mean there was a daily electronic diary that had to be completed each day for 7 days or was the reactogenicity assessment just at day 7? | Text has been updated and clarified. Page 8 – lines 141-143. |
| Page 7 line 132 - what do the authors mean by the term "validated" assay and also on line 135 by a "qualified" assay? | These words have been deleted and text has been revised to avoid any confusion. |
| Page 8 line 140. It would be helpful for readers to know that the convalescent panel comprised 29 subjects with predominantly mild disease without having to look this up in the NEJM paper. | Further review indicated that the convalescent serum panel clinical case series had been expanded since the part 1 manuscript to be inclusive of older adults. Basic description of age and clinical severity is now included in the methodology section. Page 9 – lines 166-175. |
| Statistical analysis: Page 8. There are no power calculations shown to justify the sample sizes just the assertion that with at least 150 participants per groups "there was more than adequate power to detect differences between vaccine groups" .  Can the authors provide a proper power calculation which must have been done to support the chosen trial size which was designed to compare schedules and doses to select the one to enter the Phase 3 trial. | The statement with “more than adequate” has been removed and the Statistical Analysis section has been revised – with more information on power calculations.  Page 10 – lines 177-192. |
| Results: The long narratives reporting by age group and vaccine group the various immunogenicity and reactogenicity results make very difficult reading and are really hard to take in.  Moreover there are no CIs nor numbers per group provided in the text nor are there any statements when comparing %s between groups whether any differences cited are likely to significant or not.  Why relegate all the tables to the supplementary appendix as they are a much more efficient way of communicating the results and their robustness to readers and the text need only then just summarise the key messages from that tables. | Tables have been added in manuscript text and CIs have been provided. Tables 2 and 3 in manuscript body. |
| Unsolicited adverse events: page 15. I found this confusing to read as I think that some SAEs appear multiple times under different headings - at least I hope so!  This section needs reorganising so that readers can clearly see the numbers of occurrences of the events mentioned, irrespective of what category they come under in the protocol definition. | We have rewritten the paragraphs on SAEs in particular, partially addressing the comment – but note that we need to report the AEs by category, eg. SAEs, PIMMC, etc. Pages 22-23, lines 336-354. |
| Page 17: line 296 - this speculation about immunosenescence belongs in the discussion not the results. | This statement was removed from Results section. |
| Discussion Line 370. The authors state that the neutralising antibodies were similar to or exceeded those "in a small sample of convalescent sera from hospitalised patients".  In fact looking at the NEJM paper in which information on this sample is provided it seems that there are only 4 sera among the 29 that are from hospitalised patients and they had a GMT of 7457 which vastly exceeds that shown in Table S9 for the 49 individuals aged 18-84 years with post dose 2 neutralising titres in both the low and high dose groups.  Can the authors provide the evidence for their statement?. | As described above, the details of the clinical case series reported in Figure 3b have been revised and conclusive statements rewritten. Page 9 – lines 166-175 for clinical case series info, and statement in discussion page 28, lines 470-473. |
| The authors say they have no conflicts of interest but I note that all are employed by Novavax. | Disclosure forms have been provided by the authors and will be available with the full text of this article at the *PLOS Medicine* website.  We do not believe that conflicts of interest statements for employed persons that are clearly referenced in authorship requires separate declaration as conflicts of interest since employment is clearly indicated. |
| **Comments from Reviewer 3 (Expert Independent Reviewer)** |  |
| I appreciated the opportunity to review this paper on safety and immunogenicity of the NVX-CoV2373 vaccine in younger and older adults. Overall it is a clearly written paper which certainly makes a useful contribution to the evolving literature on COVID 19 vaccines. The methods are rigorous and well-described. Validated or qualified laboratory assays were used. The figures presenting the safety and immunogenicity data are clear. |  |
| 1. Methods  a)please specify what placebo was used  b)what was the follow up period for SAE reports presented here? | Tables have been updated with 95% CI where available and Methods section has been updated. Locations as before.  We chose CIs instead of p-values because p-values only indicate whether a result is statistically significant whereas CIs also tell the strength and direction of the significance.  0.9% sodium chloride placebo has been added to Methods section. Page 7 – line 125.  December 9 date was added in safety section to state the time until which SAEs were followed up. Page 18 – line 234. |
| 2. Results  a)the mean age of the 60-84 year old participants was approx 67 between groups. The authors should discuss that this study cohort does not reflect the elderly population most at risk of severe COVID.  Please also provide data on co-morbidties in this group e.g. cardiovascular disease, COPD to show how these results can be extrapolated to the frail elderly. | We have added 75-84 year old age details. (Table 1 – page 12, line 206.  Also, we have added a statement on limitations of our study with regard to the elderly, particularly those over 85 years of age. Page 29 – lines 483-487.  As for co-morbidities, we did not have robust measurements of severity of co-morbid conditions, only the self-reported prior medical history; inclusion/exclusion had to be considered.  Nevertheless, we state in the manuscript: “…this phase 2 study was more permissive of inclusion of persons with clinically stable co-morbidities than would normally be the case for a phase 2 study, given the pandemic situation to broaden the generalizability of the result.” Page 29 – lines 485-487. |
| b)line 206 - do you mean' For vaccine participants who received placebo as the second injection, frequencies of solicited local adverse events were similar to participants who received a single dose of placebo'? | We revised these paragraphs to provide more clarification. Page 20 – lines 283-285. |
| c)please clarify whether grade 4 potentially life threatening events are counted as SAEs (line 189). It appears that ER attendance does not count as a SAE but hospitalisation does. These definitions could be included in the supplementary material. | Statement was added that no solicited grade 4 AEs met seriousness criteria for SAEs. Page 22 – lines 333-334. |
| d) please comment on 1.3% seroconversion in placebo group (line 280). Was this due to lack of specificity in the assay or asymptomatic disease in the cohort? | It is presumed that there was a low level of asymptomatic or untested mild clinical disease that led to the low level of sero-conversion. |
| Was this confirmed by N protein seroconversion? | N protein seroconversion was not done for this phase 2 study. |
| e) Please specify if any paticipants developed COVID -19 during the period described | The CONSORT diagram shows the small numbers that dropped out of immunogenicity analysis sets for all reasons. We have added a statement that positive COVID test at screening or prior to testing at a particular timepoint were excluded from the PP Analysis Set for immunogenicity at that timepoint in the Methods Immunogenicity section. Page 10 – lines 189-192. |
| f) please specify when recruitment occurred (month, year) | Recruitment date is reported in the trial results paragraph at the start of Results section: Page 10 – lines 195-197.  “Between August 24, 2020, and September 25, 2020, a total of 1288 participants underwent randomization at 17 sites in Australia and the United States in the phase 2 component of the trial.” |
| g) It would be useful to see comparative statistics on ELISA/neutralising tires between first and second doses at each dosing regime and between ages. | Immunogenicity tables by treatment group and age group with CIs are now presented in tabular format in the manuscript (Tables 2 and 3), and the immunogenicity results section has been rewritten and simplified referencing these data. Pages 23-25.  Comparative statistics were not part of the SAP for this study and will not be performed; however, the easier access to CIs for immunogenicity estimates for both assays by age group are informative about the precision of the estimates and the degree to which CIs may overlap or be non-overlapping. |
| 3. Discussion  a) The authors should set the context for their findings by considering other deployed vaccines that have looked at immunogenicity and reactogenicity in adults of different ages. E,g, Gamelaya, CanSino, AZ, Moderna. E.g. in contrast to the authors' findings, the AZ vaccine is less reactogenic in elderly adults. | Section added to Discussion referencing data from other vaccines – refs are also provided. Pages 29-30 – lines 488-498.  NVX-CoV2373 is less reactogenic in elderly adults, so it is not clear what the reviewer means here in relation to “in contrast to the authors findings.” |
| b)The authors should acknowledge that in the absence of a correlate of protection, the significance of absolute binding or neutralising antibodies is unclear. | We added a new table that partially addresses this concern with inclusion of assay results from a convalescent serum panel - Table 3 – Microneutralization Immune Responses (MN_50_) for NVX-CoV2373 Study Treatments by Study Group and Age Subgroup (18 to 59 Years and 60 to 84 Years).  We recognize the lack of immune persistence data as a limitation and have included a statement to this effect. Page 29 – lines 481-482. |
| T cell immunity may also be important but is not presented here. | T-cell immunity is not discussed in our current manuscript, but we intend to look at it for future publications, which is mentioned in the manuscript. |
| They should also refer to the press-released Novavax efficacy data to link how the observed seroconversion may translate into protection from disease. | Peer-reviewed reference for the SA study was added and the unique identification code from clinical trials.gov (NCTs) of other clinical endpoint studies are included. Novavax does not provide reference to press releases in peer-reviewed manuscripts, and individuals can use the NCTs to check for publications at a later time. |
| 4. Figures  a) Fig 1 please show number of participants who received dose 1 and who went on to receive dose 2. | This can be seen in the revised CONSORT diagram. Figure 1 – page 38, line 639 |
| Please also clarify 'early termination' - is this study withdrawal? Is this the same as 'discontinued intervention'? | The revised CONSORT diagram gives more detailed information on who withdrew or discontinued treatment. Referral to ”early termination” has been removed and instead just “study discontinuation” has been used instead. Figure 1 – page 38, line 639 |
| 5. Supplementary  a)Please present percentages of adverse events together with absolute numbers in the tables | This has been done in the safety tables included in supplementary materials. (Tables S5a – 5c, pages 12-28 of supplement) |
