# Supplementary material for: Different dose regimens of a SARS-CoV-2 recombinant spike protein vaccine (NVX-CoV2373) in younger and older adults: A phase 2 randomized placebo-controlled trial
Source: PLoS Med. 2021 Oct 1;18(10):e1003769. doi: 10.1371/journal.pmed.1003769 (PMC8486115; doi:10.1371/journal.pmed.1003769)
Supplement: Attachment — Submitted filename: Re-review_21-01479R1.docx [file pmed.1003769.s005.docx]

**Re-review of ‘Evaluation of a SARS-CoV-2 vaccine NVX-CoV2373 in younger and older adults’**

The authors have provided suitable responses to the majority of my comments. Some remaining issues are noted below, the most substantive concerns are over remaining statements that are not supported by their analyses.

The manuscript has been improved by the inclusion of point estimates and 95%CIs in the text and in Tables 2-3. The explanation for the omission of p-values is perfectly acceptable. The text added to Statistical Analysis section on power calculations and clarifying the basis of the decision to move forward to late-stage testing was also helpful. However, several statements remain about higher/lower responses throughout the manuscript that need to be amended if they cannot be supported by formal statistical inference. Most notably the last sentence of the abstract that states: “In addition, in older adults, the two-dose regimen of 5 μg was also well tolerated and ***as immunogenic*** as the two-dose regimen of 25 μg, supporting use of the lower-dose regimen in late-phase efficacy studies.” The “as immunogenic” phrase implies that non-inferiority testing was done to formally establish this. Suggest changing this to “showed sufficient immunogenicity to support its use in late-phase efficacy studies”.

In addition, the study limitations should include mention of the lack of formal statistical inference to assess superiority or non-inferiority of the two vaccine regimens.

Minor typo on line 196: “sis Subset”
