# Supplementary material for: Different dose regimens of a SARS-CoV-2 recombinant spike protein vaccine (NVX-CoV2373) in younger and older adults: A phase 2 randomized placebo-controlled trial
Source: PLoS Med. 2021 Oct 1;18(10):e1003769. doi: 10.1371/journal.pmed.1003769 (PMC8486115; doi:10.1371/journal.pmed.1003769)
Supplement: Attachment — Submitted filename: PMEDICINE-D-21-01479R2_Letter_2ndResponseSet_072821.v2.docx [file pmed.1003769.s006.docx]

Beryne Odeny, MD, MPH
*PLOS Medicine*

July 28, 2021

Dear Dr. Odeny:

Thank you for the second set of comments on our manuscript, now with the revised title “Different dose regimens of a SARS-CoV-2 recombinant spike protein vaccine (NVX-CoV2373) in younger and older adults: A phase 2 randomized placebo-controlled trial” (PMEDICINE-D-21-01479R2). We have reviewed the comments, and we have provided point-by-point responses to the comments in the table below.

According to your instructions, we have submitted two versions of the manuscript revision – a clean version and a marked-up version (tracked), which shows the changes we have made. We have also provided a revised CONSORT checklist (with sections and paragraphs, as requested) and a revised supplementary appendix that has the revised manuscript title.

Thank you for considering our manuscript for publication in *PLOS Medicine.*

Sincerely,

Neil Formica, MBBS

On behalf of the authors

**Second review of “Evaluation of a SARS-CoV-2 vaccine NVX-CoV2373 in younger and older adults”**

**PMEDICINE-D-21-01479R2 [pages and line numbers are from the clean version of manuscript]**

| **FEEDBACK:** | **RESPONSES** |
| --- | --- |
| **PLOS MEDICINE Editorial Reviewers** |  |
| 1) Please revise your title, include setting, and place the study design ("A phase II randomized placebo-controlled trial,") in the subtitle after a colon. For example, “Different dose regimens of a SARS-CoV-2 recombinant spike protein vaccine (NVX-CoV2373) in younger and older adults in USA: A phase 2 randomized placebo-controlled trial” | The title has been revised:  **Different dose regimens of a SARS-CoV-2 recombinant spike protein vaccine (NVX-CoV2373) in younger and older adults: A phase 2 randomized placebo-controlled trial** |
| 2) Author summary: please use bullet point(s) under the subheading, “What do these finding mean?” | Bullet point has been added to the Author Summary [page 4, lines 69-70] under the subheading “What do these findings mean?” [Note: made “findings” plural] |
| 3) In the abstract, please include the setting/ location of the trial. | The following statement was added to the Abstract to show setting/location [page 2, lines 28-29]: “The trial was conducted at nine sites in Australia and eight sites in the United States.” |
| 4) Please include p-values in addition to 95% CIs in both the main text (including abstract) and tables. | As stated in our initial comments, we chose CIs instead of p-values because p-values only indicate whether a result is statistically significant whereas CIs also tell the strength and direction of the significance. It was also stated in the re-review document provided from reviewers with the acceptance notification that “The explanation for the omission of p-values is perfectly acceptable.” |
| 5) Thank you for providing your CONSORTchecklist. Please replace the page numbers with paragraph numbers per section (e.g. "Methods, paragraph 1"), since the page numbers of the final published paper may be different from the page numbers in the current manuscript. | CONSORT checklist page numbers have been replaced with section and paragraph numbers. |
| 6) There are still instances of the term “subject” in the main text. Please replace "subject" with participant, patient, individual, or person. | The term “subject” has been replaced with “participant” in all instances. |
| 7) The terms gender and sex are not interchangeable (as discussed in [http://www.who.int/gender/whatisgender/en/](https://urldefense.com/v3/__http:/www.who.int/gender/whatisgender/en__;!!IfJP2Nwhk5Z0yJ43lA!eIACoBZgpsVGrMrAJ4LAiy4SYVHsiLH1CBKOdP01uBkReEut_1DoU4EKiDslgsl_QRKG$)); please use the appropriate term. | The term “gender” has been replaced by “sex” in the appropriate place in the manuscript [page 25, line 400]. |
| **PLOS MEDICINE Reviewer [separate document]** |  |
| The authors have provided suitable responses to the majority of my comments. Some remaining issues are noted below, the most substantive concerns are over remaining statements that are not supported by their analyses. The manuscript has been improved by the inclusion of point estimates and 95%CIs in the text and in Tables 2-3. The explanation for the omission of p-values is perfectly acceptable. The text added to Statistical Analysis section on power calculations and clarifying the basis of the decision to move forward to late-stage testing was also helpful. | We are pleased that the reviewer found our responses to their comments suitable. |
| However, several statements remain about higher/lower responses throughout the manuscript that need to be amended if they cannot be supported by formal statistical inference. Most notably the last sentence of the abstract that states: “In addition, in older adults, the two-dose regimen of 5 μg was also well tolerated and ***as immunogenic*** as the two-dose regimen of 25 μg, supporting use of the lower-dose regimen in late-phase efficacy studies.” The “as immunogenic” phrase implies that non-inferiority testing was done to formally establish this. Suggest changing this to “showed sufficient immunogenicity to support its use in late-phase efficacy studies”. | We have revised the last statement in the abstract as suggested by the reviewer [page 3, lines 51-53]:  “In addition, in older adults, the two-dose regimen of 5 µg was also well tolerated and showed sufficient immunogenicity to support its use in late-phase efficacy studies.” |
| In addition, the study limitations should include mention of the lack of formal statistical inference to assess superiority or non-inferiority of the two vaccine regimens. | We have added a statement with similar wording as suggested by the reviewer in the Discussion [page 29, lines 499-500]:  “Another limitation was the lack of formal statistical inference to assess superiority or non-inferiority of the two vaccine regimens.” |
| Minor typo on line 196: “sis Subset” | We have fixed the typo [page 10, line 197]. It now reads as “This Subset” |
| **Reviewer #3** |  |
| Thank you for addressing my queries, I have no further comments. | We thank Reviewer #3 for the review. |
